# Large-Scale Molecular Evolutionary Analysis Uncovers a Variety of Polynucleotide Kinase Clp1 Family Proteins in the Three Domains of Life

**DOI:** 10.1093/gbe/evz195

**Published:** 2019-09-12

**Authors:** Motofumi Saito, Asako Sato, Shohei Nagata, Satoshi Tamaki, Masaru Tomita, Haruo Suzuki, Akio Kanai

**Affiliations:** 1 Institute for Advanced Biosciences, Keio University, Tsuruoka, Japan; 2 Systems Biology Program, Graduate School of Media and Governance, Keio University, Fujisawa, Japan; 3 Faculty of Environment and Information Studies, Keio University, Fujisawa, Japan

**Keywords:** protein family, multidomain protein, experimental verification, molecular evolution, large protein, comprehensive identification

## Abstract

Clp1, a polyribonucleotide 5′-hydroxyl kinase in eukaryotes, is involved in pretRNA splicing and mRNA 3′-end formation. Enzymes similar in amino acid sequence to Clp1, Nol9, and Grc3, are present in some eukaryotes and are involved in prerRNA processing. However, our knowledge of how these Clp1 family proteins evolved and diversified is limited. We conducted a large-scale molecular evolutionary analysis of the Clp1 family proteins in all living organisms for which protein sequences are available in public databases. The phylogenetic distribution and frequencies of the Clp1 family proteins were investigated in complete genomes of Bacteria, Archaea and Eukarya. In total, 3,557 Clp1 family proteins were detected in the three domains of life, Bacteria, Archaea, and Eukarya. Many were from Archaea and Eukarya, but a few were found in restricted, phylogenetically diverse bacterial species. The domain structures of the Clp1 family proteins also differed among the three domains of life. Although the proteins were, on average, 555 amino acids long (range, 196–2,728), 122 large proteins with >1,000 amino acids were detected in eukaryotes. These novel proteins contain the conserved Clp1 polynucleotide kinase domain and various other functional domains. Of these proteins, >80% were from Fungi or Protostomia. The polyribonucleotide kinase activity of *Thermus scotoductus* Clp1 (*Ts*-Clp1) was characterized experimentally. *Ts*-Clp1 preferentially phosphorylates single-stranded RNA oligonucleotides (*K*m value for ATP, 2.5 µM), or single-stranded DNA at higher enzyme concentrations. We propose a comprehensive assessment of the diversification of the Clp1 family proteins and the molecular evolution of their functional domains.

## Introduction

Polynucleotide kinases (PNKs) catalyze the transfer of a monophosphate from a nucleoside triphosphate (NTP; usually ATP) to the 5′ end of either RNA or DNA, and the kinase module belongs to the P-loop phosphotransferase superfamily (Wang et al. 2002; Weitzer and Martinez 2007). PNKs are involved in important cellular events, including DNA repair (Weinfeld et al. 2011; Tahbaz et al. 2012; Chalasani et al. 2018), RNA processing (Weitzer and Martinez 2007; Ramirez et al. 2008; Holbein et al. 2011), and RNA repair (Wang et al. 2002; Zhang et al. 2012; Das et al. 2013). They often contain other functional domains, such as phosphatase, RNA ligase, or cyclic phosphodiesterase (CPDase) domains. For example, bacteriophage T4 polynucleotide kinase/phosphatase (T4 PNKP) is a bifunctional enzyme with 5′-OH kinase and 3′-phosphatase activities, and phosphorylates either RNA or DNA in the corresponding repair pathways (Wang et al. 2002; Zhu et al. 2007). T4 PNK is used to end-label RNA or DNA in molecular biology applications (Chaconas and van de Sande 1980). Another example is a mammalian polynucleotide kinase/phosphatase (mPNKP) that creates 5′-phosphate and 3′-hydroxyl termini for ligation in the DNA repair pathway (Bernstein et al. 2005; Bernstein 2009). Bacterial *Clostridium thermocellum* PNKP contains three catalytic domains, a PNK domain in the N-terminal region, a phosphatase domain in the central region, and an RNA ligase domain in the C-terminal region, and is involved in the RNA repair pathway (Martins and Shuman 2005; Wang et al. 2012; Zhang et al. 2012). The plant and fungal tRNA ligase, Trl1, contains three catalytic domains (Wang and Shuman 2005; Wang et al. 2006; Englert et al. 2010): An RNA ligase domain in the N-terminal region, a PNK domain in the central region, and a CPDase domain in the C-terminal region. Yeast Trl1 is reported to be an essential enzyme in RNA repair, noncanonical premRNA splicing, and pretRNA splicing (Phizicky et al. 1986; Ruegsegger et al. 2001; Schwer et al. 2004).

An enzymatic activity that specifically phosphorylates RNA molecules was detected in HeLa nuclear extracts approximately 40 years ago (Shuman and Hurwitz 1979). The corresponding enzyme was recently identified as Clp1, which was initially extensively characterized in yeast as a component of the mRNA 3′-end cleavage and polyadenylation factor complex (Minvielle-Sebastia et al. 1997; Gross and Moore 2001; Weitzer and Martinez 2007; Holbein et al. 2011). In 2000, the factors required for endonucleolytic cleavage and polyadenylation during 3′-end formation in mammalian premRNAs were purified from HeLa nuclear extracts. *Homo sapiens* Clp1 (*Hs*-Clp1) was one of these factors and was shown to be essential for the 3′-end cleavage of mRNA but not for its polyadenylation (de Vries et al. 2000; Paushkin et al. 2004). Purified *Hs*-Clp1 also has RNA kinase activity and the enzyme is involved in the pretRNA splicing reaction (Weitzer and Martinez 2007). *Hs*-Clp1 also forms a complex with the tRNA splicing endonuclease (TSEN), a multisubunit enzyme involved in the removal of tRNA introns from pretRNAs (Hanada et al. 2013; Weitzer et al. 2015). It should be noted that most tRNA introns in eukaryotes are located within the anticodon loop (canonical position) of the pretRNA. However, in Archaea, many tRNA introns are also located at other sites (noncanonical positions) (Sugahara et al. 2008; Fujishima et al. 2010). Moreover, the precursor sequences of split tRNAs, which have currently only been found in archaeal species, show high sequence similarity to the tRNA intron sequences in related archaeal species (Fujishima et al. 2009). Therefore, all these pretRNAs in Archaea are essentially spliced by the same mechanism as those in eukaryotes (Yoshihisa 2014). Many protein factors involved in pretRNA splicing have common characteristics. However, the archaeal system is much simpler than the eukaryotic system. For example, human RtcB tRNA ligase binds to a set of proteins as subunits (Popow et al. 2014), whereas archaeal RtcB requires no proteins except Archaease (Desai et al. 2014).

Clp1 is basically conserved in many eukaryotic species, including *Homosapiens* (*Hs*), *Mus musculus* (*Mm*), *Caenorhabditis elegans* (*Ce*), *Drosophila melanogaster* (*Dm*), *Arabidopsis thaliana* (*At*), *Schizosaccharomyces pombe* (*Sp*), and *Saccharomyces cerevisiae* (*Sc*) (Xing et al. 2008). It has been shown experimentally that both purified *Hs*-Clp1 and *Ce*-Clp1 phosphorylate RNA and weakly phosphorylate DNA (Weitzer and Martinez 2007; Dikfidan et al. 2014). It has also been reported that kinase-dead Clp1 mice accumulated a set of small RNA fragments derived from the aberrant processing of tyrosine pretRNA and that these fragments induced TP53-dependent cell death, resulting in the progressive loss of spinal motor neurons (Hanada et al. 2013). However, *Sc*-Clp1 has no kinase activity because it has accumulated mutations in its PNK domain (Ramirez et al. 2008; Dikfidan et al. 2014). Structurally, the eukaryotic Clp1 enzymes consist of three functional domains: The N-terminal domain, PNK domain, and C-terminal domain (Noble et al. 2006). Because the PNK activity is lost if either the N-terminal or C-terminal domain is deleted, both domains are important for the maintenance of its PNK activity (Dikfidan et al. 2014). The PNK domain contains four conserved motifs (Leipe et al. 2003; Orelle et al. 2003; Pillon et al. 2018): 1) the Walker A motif or phosphate-binding loop (P-loop), GxxxxGK[S/T], which is an NTP-binding motif; 2) the Walker B motif, [D/E]hhQ (h is a hydrophobic residue), in which the conserved aspartic acid residue is required for the coordination of divalent cations and its catalytic activity; 3) the Clasp motif, [T/S/L]xGW, which is important for RNA binding; and 4) the Lid motif, RxxxxR, which is required for ATP binding and to stabilize the transition state of the phosphotransferase reaction. There is a Clp1-related enzyme in Archaea, and purified *Pyrococcus horikoshii* Clp1 (*Ph*-Clp1) has thermostable 5′-OH PNK activity for the 5′-OH ends of both RNA and DNA, although it prefers RNA in a competitive situation (Jain and Shuman 2009). The eukaryotic PNKs, Nol9, and Grc3, also share similarities with Clp1. These enzymes phosphorylate both RNA and DNA, and are involved in prerRNA processing (Braglia et al. 2010; Heindl and Martinez 2010). It has also been reported that some proteins in Bacteria share similarities with the Clp1 PNK domain (Xing et al. 2008). In this paper, we regard all these proteins as members of the Clp1 family of enzymes. Until now, Clp1 and its family of enzymes have been characterized and reported separately for all three domains of life, and there is no comprehensive evolutionary analysis of the Clp1 family enzymes (Leipe et al. 2003). Nor is the detailed evolutionary scenario that accounts for their overall diversity fully understood.

Therefore, in this study, we conducted a large-scale molecular evolutionary analysis of the Clp1 family proteins in the three domains of life and propose a possible evolutionary scenario for them. During this research, we also found a group of large proteins, each containing the conserved Clp1 PNK domain. Finally, we provide the first experimental evidence that a bacterial Clp1 protein from *Thermus scotoductus* (*Ts*-Clp1) shows PNK activity.

## Materials and Methods

### Data Sources

To undertake a large-scale search for Clp1 family proteins in the three domains of life, a total of 137,772,056 coding sequences (CDSs) were obtained from the UniProtKB database (December 2018 data set) at ftp://ftp.uniprot.org/pub/databases/uniprot/current_release/; last accessed September 17, 2019. (table 1*A*; The UniProt 2017). In a detailed evolutionary analysis of the Clp1 family proteins in the complete genomes of prokaryotes (5,468,108 CDSs) and eukaryotes (8,534,278 CDSs), together with their species information, were obtained from the Reference Sequence (RefSeq) database (Prokaryotes, August 2018 data set, and Eukaryotes, August 2019 data set) at ftp://ftp.ncbi.nlm.nih.gov/genomes/refseq/; last accessed September 17, 2019. (table 1*B*; O'Leary 2016). Seventy-two representative species (36 bacterial, 18 archaeal, and 18 eukaryotic species), each with a complete genomic sequence, were randomly selected according to a previous report (Ciccarelli et al. 2006). For the domain analysis, 16,712 domains, together with their reliable annotations, were obtained from the Pfam-A database (version 32) at ftp://ftp.ebi.ac.uk/pub/databases; last accessed September 17, 2019. (Finn et al. 2014). The taxonomic classification was performed with the NCBI Taxonomy Database (https://www.ncbi.nlm.nih.gov/taxonomy; last accessed September 17, 2019).

### Sequence Similarity Search for Clp1 Family Proteins

To comprehensively identify Clp1 family proteins in both the UniProt KB and RefSeq databases, a protein–protein BLAST (BLASTP, ver. 2.2.29+) search (Boratyn et al. 2013) was performed with an *E*-value of ≤1e − 4 and query coverage of ≥70%. We used several query sequences (supplementary table S1, Supplementary Material online) to cover the diverse Clp1 family proteins, including Clp1-related proteins such as Nol9 and Grc3 in eukaryotes and a bacterial Clp1 protein that was originally annotated as a GTPase in the database. “Amino acid identity” was defined as the percentage of amino acid [aa] residues in two different sequences that were identical. “Amino acid similarity” was defined as the percentage of identical or similar aa residues, based on similar physicochemical properties. We used both identity and similarity scores calculated with the BLAST program using the BLOSUM62 matrix (Henikoff and Henikoff 1992).

### Amino Acid Sequence Alignment, Domain Search, and Phylogenetic Tree

The aa sequences of the Clp1 family proteins were aligned with MAFFT version 7.394, with the default parameters (Katoh and Standley 2013). The multiple-sequence alignment was used to construct a phylogenetic tree using the GTR model with the RAxML software version 8.2.11 (Stamatakis 2014). The results were visualized with either Jalview (version 2.10.3) (Waterhouse et al. 2009) or SeaView (version 4.5.4) (Gouy et al. 2010). To extract the protein domains, an HMMER (ver. 3.2) search (Potter et al. 2018) of the Pfam-A protein domain database was performed with an *E*-value of ≤1e − 4. The domain structures and sequence alignment of the Clp1 family proteins were visualized with DoMosaics (version rv0.95) (Moore et al. 2014).

### Construction of an Expression Vector for *T. scotoductus* Clp1 (*Ts*-Clp1)

To efficiently produce the recombinant *Ts*-Clp1 protein (UniProt accession number [AC]: E8PQM6) in *Escherichiacoli*, a synthetic gene was designed to optimize codon usage with a WEB tool provided by Eurofins Genomics Tokyo (https://www.eurofinsgenomics.jp; last accessed September 17, 2019; see also supplementary fig. S9, Supplementary Material online). The synthetic *Ts*-*clp1* gene was designed to contain *Nde*I and *Xho*I sites at its 5′ and 3′ termini, respectively, and was subcloned into these restriction sites in the pET-23b expression vector (Novagen, Madison, WI, USA). The resulting pET-*Ts*-Clp1 vector encoded a protein with a six-histidine (His) tag at its C-terminal end.

### Expression and Purification of His-Tagged Recombinant *Ts*-Clp1 Protein

To express the recombinant *Ts*-Clp1 protein, *E. coli* strain BL21(DE3) was transformed with the expression vector pET-*Ts*-Clp1. The transformants growing logarithmically at 37 °C in Luria–Bertani (LB) medium containing 50 µg/ml ampicillin were treated with 0.4 mM isopropyl-β-d-thiogalactoside (IPTG). After further growth for 16 h at 30 °C, the cells were harvested by centrifugation (9,000 × g for 15 min at 4 °C), and the protein was extracted with sonication (3–4 min) in His-tag-binding buffer containing 20 mM Tris–HCl (pH 8.0), 500 mM NaCl, 5 mM imidazole, and 0.1% (v/v) NP-40. The extract was heat-treated at 60 °C, the growth temperature of *T. scotoductus*, for 15 min to destroy any endogenous *E. coli* proteins and then centrifuged at 18,000 × g for 10 min at 4 °C to remove any debris. The recombinant protein was purified with a HisTrap HP column (GE Healthcare, Piscataway, NJ, USA) and eluted with a linear gradient of imidazole (5–1,000 mM) in His-tag-binding buffer using the AKTA, fast protein liquid chromatography (FPLC) System (GE Healthcare). The eluted protein peak was collected and dialyzed against buffer D containing 50 mM Tris–HCl (pH 8.0), 1 mM ethylenediaminetetraacetic acid (EDTA), 0.02% (v/v) Tween 20, 7 mM 2-mercaptoethanol, and 10% (v/v) glycerol.

### PNK Assay

The PNK activity was assayed by analyzing a fluorescein amidite (FAM)-labeled oligoribonucleotide probe on a 15% (w/v) polyacrylamide gel containing 8 M urea, because we have previously demonstrated that the migration of the oligoribonucleotide under these conditions varies with the terminal phosphate structure (Kanai et al. 2009; Sato et al. 2011). Basically, the reactions were performed in 20 μl of reaction buffer containing 20 mM Tris–HCl (pH 8.0), 1 mM DTT, 50 mM KCl, 10 mM MgCl_2_, 1 mM ATP, 25 picomoles of 5′-R20–FAM-3′, and purified recombinant *Ts*-Clp1 (0.5 μg/ml). After the samples were incubated for 15 min at 60 °C, we added an equal volume of stop solution (8 M urea, 1 M Tris–HCl [pH 8.0], and a small amount of Blue Dextran [Sigma Chemical, St. Louis, MO, USA]) to stop the reactions. The reaction mixtures were heated at 70 °C for 5 min, loaded onto a 15% (w/v) polyacrylamide gel containing 8 M urea, and run for 20 min at 1,600 V and for 90 min at 1,800 V. The reaction products were visualized with a Molecular Imager FX Pro (Bio-Rad Laboratories, Hercules, CA, USA). To determine the kinetic parameters of the enzyme, the ATP concentration was varied from 0.1 to 50 µM. The *K*m value was calculated from a Lineweaver–Burk plot. The sequences of the oligoribonucleotides used in this assay are summarized in supplementary table S8, Supplementary Material online.

## Results

### Extraction and Distribution of Clp1 Family Proteins in the Three Domains of Life

To detect the Clp1 family proteins (Clp1 or Clp1-related proteins) on a large scale, several PNK domain regions of representative Clp1 proteins were used as query sequences (supplementary table S1 and supplementary fig. S1, Supplementary Material online). For bacterial Clp1 proteins, in the first round of extraction, in which we used the PNK domain of archaeal Clp1 (UniProt AC: O57936) as the query sequence, a bacterial Clp1 protein was identified that had been annotated a GTPase or GTP-binding protein (UniProt AC: A5G778 in supplementary table S1, Supplementary Material online). We selected this as the query sequence for a comprehensive second round of extraction for bacterial Clp1 proteins. In summary, a BLASTP search (*E*-value ≤ 1e − 4) was performed against the UniProtKB database (release: December 2018), which contains 137,772,065 proteins (table 1*A*). As a result, 3,557 Clp1 family protein sequences were obtained (the list contains those proteins detected with a metagenomic analysis and proteins with partial Clp1 aa sequences). It should be noted that T4 PNK, which is known to phosphorylate the 5′ ends of DNA and RNA (Wang et al. 2002), was not extracted under our search conditions.

**Table 1:** Data Sets Used in This Study

(A) Numbers of CDSs in the three domains of life from the UniProtKB database are shown					
	Eukarya	Bacteria	Archaea	Others	Total
Species	1,259,362	486,584	13,015	193,814	1,952,775
CDS	32,407,431	96,898,645	2,980,127	5,485,853	137,772,065


(B) Numbers of CDSs in the three domains of life determined from complete genomes in the RefSeq database are shown			
	Eukarya	Bacteria	Archaea
Species	288	1,543	140
CDS	8,534,278	5,131,344	336,764


**Fig. 1.**
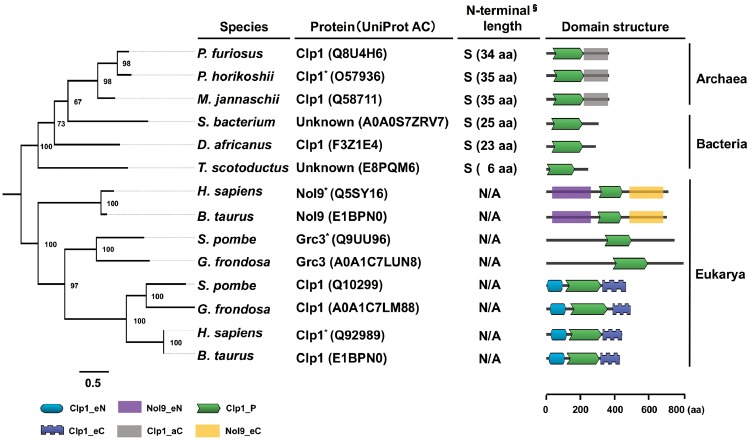
—Phylogeny and domain structure of Clp1 family proteins in the three domains of life. Phylogenetic trees were constructed based on the amino acid sequences of 14 selected Clp1 family proteins using the maximum likelihood method with 1,000 bootstrap replicates. Numbers on the branches indicate bootstrap values. Scale bar under the tree indicates the number of amino acid substitutions per site. Because there is no exact outgroup for the three domains of life, midpoint rooting was used. Proteins that were experimentally characterized are marked with an asterisk. In this study, we demonstrated the polynucleotide kinase activity of a bacterial protein from *Thermus scotoductus* (UniProt AC: E8PQM6) with unknown function. Domains were visualized with DoMosaics. Domains are defined as follows: Clp1_eN, Clp1 N-terminal domain in eukaryotes; Nol9_eN, Nol9 N-terminal domain in eukaryotes; Clp1_P, polynucleotide kinase domain; Clp1_eC, Clp1 C-terminal domain in eukaryotes; Clp1_aC, Clp1 C-terminal domain in archaea; Nol9_eC, Nol9 C-terminal domain in eukaryotes. § N-terminal lengths in prokaryotic Clp1 family proteins are classified into two groups: Short (S), <60 aa; and long (L), >61 aa (see also supplementary figs. S2 and S3, Supplementary Material online). N/A, not applicable. Scale bar beneath the domain illustration shows the amino acid (aa) length of each protein. The organisms are: *Pyrococcus furiosus*, *Pyrococcus horikoshii*, *Methanocaldococcus jannaschii*, *Spirochaetes bacterium*, *Desulfovibrio africanus*, *T. scotoductus*, *Homo sapiens*, *Bos taurus*, *Schizosaccharomyces pombe*, and *Grifola frondosa*.

To classify the types of species, 3,540 of the 3,557 sequences were used because the remaining 17 sequences were annotated as “ecological metagenomes.” The species containing Clp1 family proteins are listed in supplementary table S2, Supplementary Material online. These proteins are distributed in 1,426 species of eukaryotes, 211 species of archaea, and 144 species of bacteria. In the eukaryotes, the largest number of species containing these proteins was in the Opisthokonta (1,180 species including metazoans, fungi, and protists), followed by Viridiplantae (138 species consisting of green plants). In the archaea, the largest number of these species was in the TACK superphylum (106 species, including members of the Crenarchaeota and Thaumarchaeota), followed by the Euryarchaeota (94 species). In the bacteria, the largest number of these species was in the phylum Proteobacteria (39 species), followed by the Terrabacteria group (19 species that were Gram-positive bacteria or photosynthetic bacteria). It was not possible to calculate the numbers of CDSs of Clp1 family proteins in the genomes registered in the UniProtKB database because many of these genomes were incomplete or “draft” genomes. Therefore, we counted the number of CDSs of Clp1 family proteins in the genomes of 72 representative species (36 bacterial, 18 archaeal, and 18 eukaryotic species) for which complete genomes were available (table 2), according to a previous report (Ciccarelli et al. 2006). We found Clp1 family proteins in all the representative eukaryotes (18/18; 100%) and half the archaea (9/18; 50%) (table 2*A*), but in only a limited number of bacteria (3/36; 8.3%) (table 2*B*). In the eukaryotic genomes, CDSs for Nol9 proteins were present in Metazoa (e.g., mammals, fishes, and birds) and CDSs for Grc3 were present in fungi. Both proteins are known to be involved in prerRNA processing. Bacterial proteins that were annotated as translation factor GUF1, GTPase, GTP-binding protein, or uncharacterized protein were extracted (shown as “Others” in table 2*B*), although all these proteins are essentially considered to be Clp1 family proteins (discussed below). This analysis revealed that there are two or more CDSs for Clp1 family proteins per genome in all representative eukaryotes and in some Crenarchaeota (Archaea). In plants, both *Arabidopsis thaliana* and *Oryza sativa* subsp. *japonica* have six CDSs for Clp1 family proteins. However, there is usually only one CDS for a Clp1 family protein per genome in Euryarchaeota (Archaea) and Bacteria (fig. 1 and table 2).

**Table 2:** Distribution of Clp1 Family Proteins in 72 Representative Species with Complete Genome Sequences

Taxon			Protein			
Domain	Kingdom	Species	Clp1	Nol9	Grc3	Others^a^
(A)						
Eukarya	Metazoa	*Caenorhabditis briggsae*	1	1	—	1
		*Caenorhabditis elegans*	1	1	—	—
		*Drosophila melanogaster*	1	1	—	1
		*Danio rerio*	1	1	—	—
		*Gallus gallus*	1	1	—	—
		*Pan troglodytes*	3	1	—	—
		*Homo sapiens*	2	1	—	—
		*Mus musculus*	1	1	—	—
		*Rattus norvegicus*	1	1	—	—
		*Takifugu rubripes*	1	1	—	—
		*Anopheles gambiae*	1	—	—	1
	Fungi	*Schizosaccharomyces pombe* 972	1	—	1	—
		*Saccharomyces cerevisiae* RM11-1a	1	—	1	—
		*Eremothecium cymbalariae* KCTC 17166	1	—	—	1
	Plantae	*Cyanidioschyzon merolae* 10D	1	—	—	—
		*Arabidopsis thaliana*	3	1	—	2
		*Oryza sativa subsp. japonica*	2	—	—	4
	Amoebozoa	*Dictyostelium discoideum*	1	1	—	—
Archaea	Euryarchaeota	*Methanocaldococcus jannaschii* DSM2661	1	—	—	—
		*Methanosarcina mazei* Go1	—	—	—	—
		*Methanosarcina acetivorans* C2A	—	—	—	—
		*Archaeoglobus fulgidus* DSM 8774	—	—	—	1
		*Pyrococcus furiosus* COM1	1	—	—	—
		*Thermoplasma acidophilum* DMS1728	—	—	—	—
		*Methanopyrus kandleri* DSM 6324	—	—	—	1
		*Pyrococcus abyssi* GE5	1	—	—	—
		*Methanococcus maripaludis* C6	—	—	—	—
		*Thermoplasma volcanium* GSS1	—	—	—	—
		*Pyrococcus horikoshii* OT3	1	—	—	—
		*Halobacterium salinarum* NRC-1	—	—	—	—
		*Methanothermobacter thermautotrophicus* DSM 1053	—	—	—	—
	Crenarchaeota	*Sulfolobus solfataricus* ATCC 35092	—	—	—	2
		*Pyrobaculum aerophilum* ATCC 51768	—	—	—	2
		*Aeropyrum pernix* ATCC 700893	—	—	—	1
		*Sulfolobus tokodaii* DSM 16993	—	—	—	—
	Nanoarchaeota	*Nanoarchaeum equitans* Kin4-M	—	—	—	—
(B)						
Bacteria	Firmicutes	*Lactobacillus plantarum* ATCC BAA-793	—	—	—	—
		*Clostridium acetobutylicum* ATCC 824	—	—	—	—
		*Bacillus subtilis* 168	—	—	—	—
	Planctomycetes	*Rhodopseudomonas palustris* ATCC BAA-98	—	—	—	—
		*Gemmata obscuriglobus*	—	—	—	—
	Spirochaetes	*Leptospira interrogans* 56601	—	—	—	—
		*Borrelia burgdorferi* ATCC 35210	—	—	—	—
	Actinobacteria	*Bifidobacterium longum* ATCC 15707	—	—	—	—
		*Streptomyces coelicolor* ATCC BAA-471	—	—	—	—
	Fibrobacteres	*Fibrobacter succinogenes* (s85)	—	—	—	—
	Chlorobi	*Chlorobaculum tepidum* ATCC 49652	—	—	—	—
	Bacteroidetes	*Bacteroides thetaiotaomicron* ATCC 29148	—	—	—	—
		*Porphyromonas gingivalis* ATCC 33277	—	—	—	—
	Chlamydiae	*Chlamydia trachomatis* 434/Bu	—	—	—	—
	Fusobacteria	*Fusobacterium nucleatum subsp. Nucleatum* ATCC 25586	—	—	—	—
	Thermotogae	*Thermotoga maritima* ATCC 43589	—	—	—	—
	Aquificae	*Aquifex aeolicus* VF5	—	—	—	—
	Chloroflexi	*Dehalococcoides ethenogenes* 195	—	—	—	—
	Deinococcales	*Thermus scotoductus* SA-01	—	—	—	1
		*Deinococcus radiodurans* ATCC 13939	—	—	—	—
	Cyanobacteria	*Synechocystis sp.* PCC 6803	—	—	—	—
		*Prochlorococcus marinus* MIT 9313	—	—	—	—
	Acidobacteria	*Acidobacterium capsulatum* ATCC 51196	—	—	—	—
		*Solibacter usitatus* Ellin6076	—	—	—	—
	δ-Proteobacteria	*Desulfovibrio africanus* Walvis Bay	1	—	—	—
		*Geobacter uraniireducens* Rf4	—	—	—	1
	ɛ-Proteobacteria	*Helicobacter pylori* ATCC 700392	—	—	—	—
		*Campylobacter jejuni* ATCC 700819	—	—	—	—
	α-Proteobacteria	*Agrobacterium tumefaciens* C58	—	—	—	—
		*Brucella melitensis* 16M	—	—	—	—
	β-Proteobacteria	*Bordetella parapertussis* 12822	—	—	—	—
		*Neisseria meningitidis* MC58	—	—	—	—
	γ-Proteobacteria	*Xanthomonas campestris pv. Campestris* ATCC 33913	—	—	—	—
		*Vibrio vulnificus* YJ016	—	—	—	—
		*Escherichia coli* K12	—	—	—	—
		*Buchnera aphidicola* Bp	—	—	—	—

Note.—The numbers of Clp1 or Clp1-related proteins in representative complete genomes of (A) Eukarya (18 species), Archaea (18 species), and (B) Bacteria (36 species) are shown.

a “Others” contains proteins annotated as “GTPase,” “translation factor GUF1,” or “uncharacterized protein” based on their domain similarities.

**Fig. 2.**
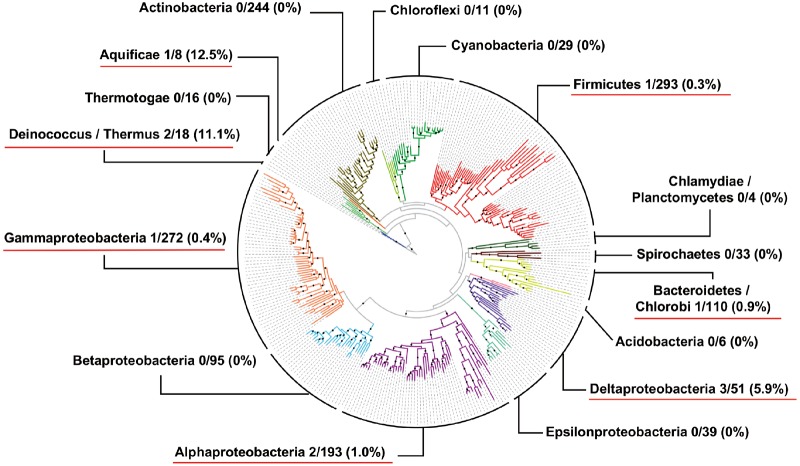
—Scattered and restricted distribution of Clp1-related proteins on bacterial phylogenetic tree. The number of bacterial Clp1-related proteins was mapped onto a bacterial phylogenetic tree consisting of 578 species with complete genome sequences (Wu and Eisen 2008). At the level of each phylum, the ratio of the number of Clp1-related proteins to the total number of species used in this study (see supplementary table S5, Supplementary Material online) is shown. The calculated ratio is also shown in parentheses. Phyla in which species possessed Clp1-related proteins are underlined in red.

The RefSeq database contains 288 complete eukaryotic genomes (release: August 2019), 1,543 complete bacterial genomes (release: August 2018), and 140 complete archaeal genomes (release: August 2018) (table 1*B*). To clarify the numbers of Clp1 family proteins per genome, a BLASTP search (*E*-value ≤ 1e − 4) of these complete genomes was performed. CDSs for Clp1 family proteins were present in 247 of the 288 species (85.8%) of Eukarya (supplementary tables S3 and S4, Supplementary Material online). In contrast, CDSs for Clp1 family proteins were present in 25.9% of Apicomplexa (parasitic alveolates) and 50.0% of Mycetozoa (slime molds). Notably, there were no CDSs for Clp1 family proteins in taxa such as Ciliophora (a group of protozoans) and Cryptophyta (unicellular flagellates) (supplementary tables S3 and S4, Supplementary Material online). CDSs for Clp1 family proteins were present in 58 of the 140 species (41.4%) of Archaea (supplementary table S5, Supplementary Material online). Because there were fewer Clp1 family proteins in Archaea, we analyzed the archaeal strains without a *clp1* gene (i.e., 26 strains of Halobacteria and 12 strains of Methanococci). We found that 23/26 (88%) Halobacteria and 9/12 (75%) Methanococci had *RtcB* genes in their genomes. Similarly, 22/26 (84%) Halobacteria and 12/12 (100%) Methanococci had *archaease* genes in their genomes. Therefore, almost all these archaeal species seem to splice their pretRNA via the RtcB-dependent ligation pathway and not via the Clp1-dependent ligation pathway. In contrast, CDSs for Clp1 family proteins were present in only 14 highly restricted species of the 1,543 species (0.9%) of Bacteria examined (supplementary table S5, Supplementary Material online). These results show that genes for bacterial Clp1 family proteins are extremely rare in bacterial genomes. To investigate the phylogenetic positions of the bacterial Clp1 family proteins, the presence or absence of these enzymes was mapped on a previously reported bacterial phylogenetic tree (Wu and Eisen 2008). Figure 2 clearly shows that the evolution of bacterial Clp1 is not lineage specific, but that it is distributed almost independently among bacteria. We also conducted a comprehensive molecular evolutionary analysis of the 235 archaeal and 149 bacterial Clp1 family proteins obtained from the UniProt Knowledgebase (UniProtKB), including both complete and incomplete genomic sequences. We found that the size distribution of the N-termini had two peaks corresponding to short and long forms (supplementary figs. 2 and 3, Supplementary Material online). The short peak mainly consisted of bacterial Clp1 proteins and the longer peak mainly consisted of archaeal Clp1 proteins. However, both peaks contained both archaeal and bacterial Clp1 proteins.

### Clp1 Family Proteins and Novel Large Proteins With Clp1 PNK Domains

As shown in figure 1, the Clp1 family proteins were divided into two major clades, the prokaryotic (archaeal and bacterial) type and the eukaryotic type, on the phylogenetic tree. The eukaryotic clade was further subdivided into three clades: Clp1, Nol9, and Grc3. The last two are involved in prerRNA processing (Braglia et al. 2010; Heindl and Martinez 2010). These results suggest that Clp1 evolved as a family of proteins with specific functions at least in certain eukaryotes. Among the protein domain structures, the PNK domain (Clp1_P) of this family of proteins is universally conserved in all three domains of life (fig. 1). Although Clp1_P is located close to the N-terminus in prokaryotes, it is located in the middle of the protein in all eukaryotic enzymes. Our research also shows that the C-terminal domain (Clp1_aC) is conserved among the archaeal Clp1 proteins (supplementary fig. S4, Supplementary Material online). Conserved N-terminal (Clp1_eN) and C-terminal (Clp1_eC) domains have been reported in the eukaryotic Clp1 proteins (Noble et al. 2006), and we also identified conserved N-terminal (Nol9_eN) and C-terminal (Nol9_eC) domains among the Nol9 enzymes (supplementary figs. S5 and S6, Supplementary Material online).

**Fig. 3.**
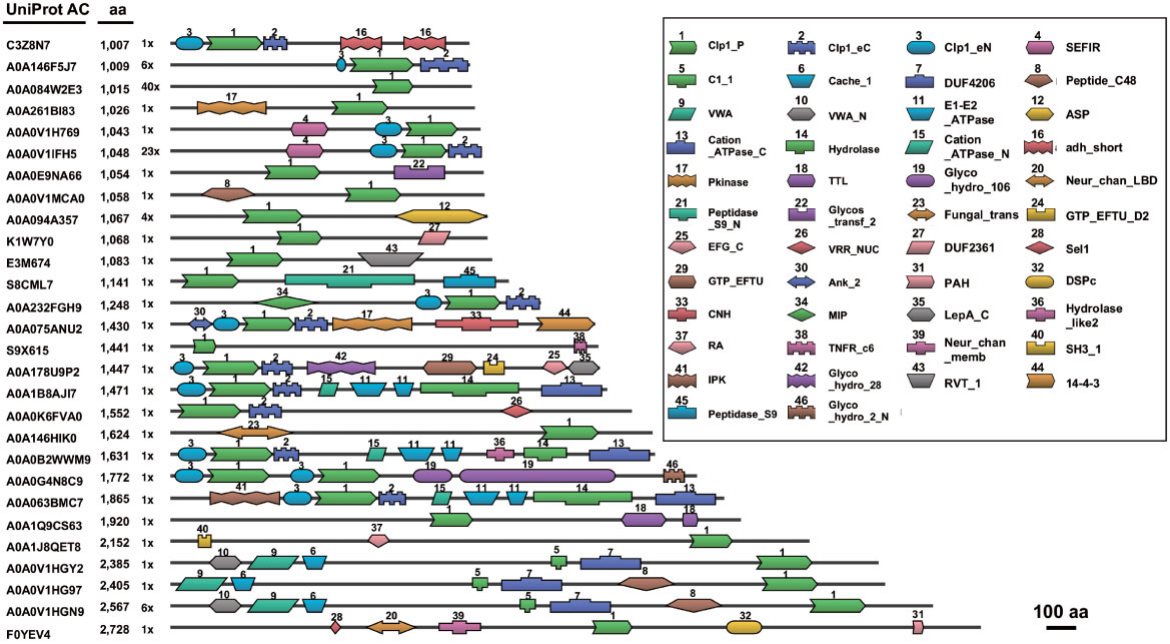
—Large proteins with the Clp1 polynucleotide kinase domain. Representative examples of large proteins that contain the polynucleotide kinase domain of Clp1 and other functional domains. Scale bar represents 100 amino acid (aa) residues. Each domain is schematically shown in a box and numbered according to its frequency of appearance. See figure 1 legend for the definition of domains Clp1_eN, Nol9_eN, Clp1_P, Clp1_eC, Clp1_aC, and Nol9_eC. Other functional domains are defined in the Pfam database (https://pfam.xfam.org/; last accessed September 17, 2019). Number of proteins with a similar domain structure is shown as “number ×.” For example, “3×” means three proteins with a similar domain structure.

To determine the size distribution of the Clp1 family proteins, 3,332 of the original 3,557 sequences were used because the remaining 225 sequences were annotated as “ecological metagenomes” and/or “fragmented sequences.” supplementary fig. S7, Supplementary Material online summarizes the size distribution of the Clp1 family proteins. The main peak is located between lengths of ∼300 and 800 aa (mean ± standard deviation, 555 ± 225 aa) and this peak contains all known enzymes, including Clp1, Nol9, and Grc3 (Braglia et al. 2010; Heindl and Martinez 2010). The smallest Clp1 (UniProt AC: M5Q339) is a 199-aa protein from a bacterium, *Desulfovibrio africanus* PCS, and contains only the PNK domain. The size distributions of the Clp1 family proteins differ across the three domains of life. In the prokaryotes, the protein lengths are rather shorter than average (376 ± 46 for archaea and 333 ± 74 for bacteria) because these Clp1 proteins lack the N-terminal domain, as described above. The eukaryotic Clp1 proteins are highly diverse in size (501 ± 226), and we detected 122 large proteins in the eukaryotes, each exceeding 1,000 aa residues (maximum length, ∼2,700 aa residues; see the inside figure of supplementary fig. S7, Supplementary Material online). All these large proteins (supplementary table S6, Supplementary Material online) contain the Clp1_P domain and many of them also contain another 1–9 functional domains (fig. 3). Among the 122 large proteins, 69 were from Protostomia, 30 were from Fungi, and 13 were from the Trypanosomatidae. Figure 3 shows representative examples of these large proteins. Several of these large proteins, such as a 1,007-aa protein (UniProt AC: C3Z8N7), a 1,009-aa protein (UniProt AC: A0A146F5J7), and a 1,048-aa protein (UniProt AC: A0A0V1IFH5), contain all the eukaryotic Clp1 domains (Clp1_eN, Clp1_P, and Clp1_eC), whereas the others usually contain the Clp1_P domain, together with other functional domains. Therefore, we conclude that the large proteins are novel proteins, possessing the whole Clp1 or partial Clp1 structure (mainly the Clp1_P domain) and other functional domains. Because some of the large proteins have similar domain architectures, such as the 1,471-aa protein (UniProt AC: A0A178U9P2) and the 1,631-aa protein (UniProt AC: A0A0B2WWM9), or the 2,385-aa protein (UniProt AC: A0A0V1HGY2) and the 2,567-aa protein (UniProt AC: A0AVIHGN9), and because some of the large proteins appear at high frequencies (e.g., 40 times for the 1,015-aa protein [UniProt AC: A0A084W2E3] and 23 times for the 1,048-aa protein [UniProt AC: A0A0V1IFH5]), many of them are not sequencing artifacts but are actually encoded in these genomes. The Clp1_P domains in the large proteins have the well-conserved motifs required for phosphorylation activity (Walker A, Walker B, and Clasp), as well as partially conserved Lid motifs (supplementary fig. S8, Supplementary Material online), suggesting that the large proteins may all have phosphorylation activity. However, the exact functions of these large proteins remain unknown. The large proteins are also annotated based on their similarities with known functional proteins. Although many of them are annotated as Clp1 homologs, Clp1-like proteins, or even uncharacterized proteins, some are annotated as specific proteins. For examples, the 1,447-aa protein (UniProt AC: A0A178U9P2) is annotated as a translation factor GUF1 homolog, the 1,552-aa protein (UniProt AC: A0A0K6FVA0) as a fanconi-associated nuclease, and the 2,385-aa protein (UniProt AC: A0A0V1HGY2) as a voltage-dependent calcium channel, unc-36. Further functional analyses are required to support these annotations (supplementary table S6, Supplementary Material online).

### Biochemical Characterization of Bacterial Clp1 Proteins

**Fig. 4.**
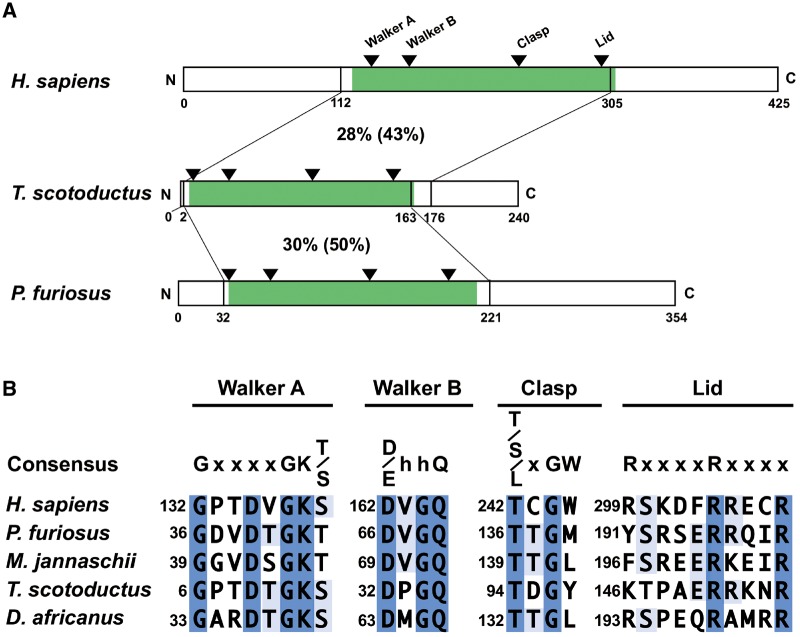
—Schematic representation of bacterial Clp1 and its conserved motifs. (*A*) Three Clp1 proteins from human (*Homo Sapiens*), bacteria (*Thermus scotoductus*), and archaea (*Pyrococcus furiosus*) are shown as bars. Numbers below the proteins refer to the positions of the amino acid residues. Percentage identities (similarities) of specific regions among the Clp1 proteins are indicated. The polynucleotide kinase domain of each protein is shown in green. The four conserved functional motifs in the polynucleotide kinase domain are indicated with triangles (see text in detail). (*B*) Amino acid sequences of the conserved motifs in the polynucleotide kinase domain were aligned with MAFFT. Amino acid sequence alignments were visualized with Jalview. Identical amino acid residues are indicated in blue and partly conserved amino acid residues are indicated in light blue. The amino acid numbers, from the first methionine (Met) residue, are shown on the left of each line. In the consensus sequence line, “x” and “h” mean any amino acid residue and a hydrophobic amino acid residue, respectively. See figure 1 legend for the five organisms used here.

Fourteen bacterial Clp1 family proteins were identified with the bioinformatic analysis, as shown in supplementary tables S5 and S7, Supplementary Material online. However, no experimental study has reported that a bacterial Clp1 protein actually has PNK activity. Therefore, our aim in this study was to demonstrate the biochemical activities of the enzyme. We investigated a bacterial Clp1 family protein (UniProt AC: E8PQM6) from *T. scotoductus*, which was isolated from a cleft in the Witwatersrand Supergroup rocks in South Africa (Gounder et al. 2011). Because the in vivo expression of the *Ts-clp1* gene was examined by RNA-Seq analysis (Cusick et al. 2016), we considered that the corresponding protein *Ts*-Clp1 must be functional. We designated the protein “*Ts*-Clp1” (based on biochemical evidence; see below), although the protein is annotated as an uncharacterized protein in the UniProt database. The *Ts*-Clp1 protein shows only 28% aa identity and 43% similarity to human Clp1 (*Hs*-Clp1), and only 30% identity and 50% similarity to *Pyrococcus furiosus* Clp1 (*Pf-*Clp1) (fig. 4*A*). At least three of the four motifs in the PNK domain (Walker A, Walker B, and Clasp) are well conserved across the three domains of life (fig. 4*B*). These three motifs are reportedly involved in phosphorylation activity (Pillon et al. 2018). Although the last motif (Lid) is less well conserved in *Ts*-Clp1, similar aa residues occur in the motifs. For example, Arg299 in human Clp1 is replaced with Lys146 in *Ts*-Clp1.

**Fig. 5.**
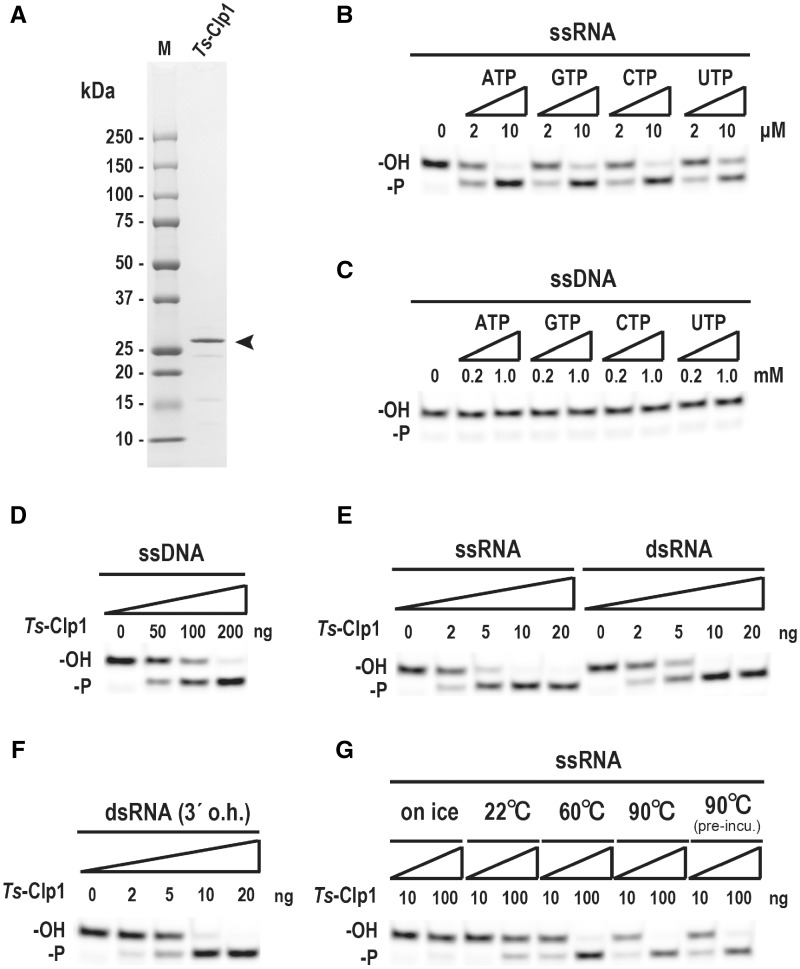
—Verification of the polynucleotide kinase activity of bacterial Clp1. (*A*) Purified recombinant *Thermus scotoductus* Clp1 (*Ts*-Clp1) was separated on 10–20% SDS-PAGE and stained with Coommassie Brilliant Blue. An arrowhead indicates the position of purified *Ts*-Clp1. (*B*–*G*) Characterization of the polynucleotide kinase activity of *Ts*-Clp1. Basically, a 3′-fluorescein amidite (FAM)-labeled oligoribonucleotide was incubated with purified *Ts*-Clp1 protein (0.5 μg/ml) and 10 mM MgCl_2_ at 60 °C for 15 min. The products were separated by 15% polyacrylamide gel electrophoresis with 8 M urea. The effects of the nucleoside triphosphate (*B*–*C*), type of nucleic acid (*D*–*F*), and temperature (*G*) on the polynucleotide kinase activity were examined. Substrates used are indicated on the top of each column. Pre-incu: The reaction mixture was preincubated at 90 °C before the enzyme was added. See also supplementary figure S10, Supplementary Material online.

For the biochemical analysis of *Ts*-Clp1 and to efficiently produce the recombinant protein in *E.coli*, we first constructed an expression vector for the *Ts-clp1* gene with codon optimization (supplementary fig. S9, Supplementary Material online). The expressed and purified His-tagged *Ts*-Clp1 had a molecular mass of approximately 27 kDa, as determined with sodium dodecyl sulfate (SDS)-polyacrylamide gel electrophoresis (PAGE) (fig. 5*A*). This finding is consistent with the size predicted from the aa sequence deduced from the corresponding gene. Because *T. scotoductus* was isolated from a South African gold mine in which the ambient temperature of the rock was ∼60 °C (Kieft et al. 1999), the PNK activity of *Ts*-Clp1 (10 ng/reaction) was examined at 60 °C for 15 min in the presence of NTPs and MgCl_2_. Under these conditions, *Ts*-Clp1 phosphorylated a single-stranded RNA (ssRNA) oligonucleotide in the presence of 2–10 µM NTPs (*K*m for ATP: 2.5 µM) (fig. 5*B* and supplementary fig. S10, Supplementary Material online). *Ts*-Clp1 also uses ATP in preference to other NTPs: The relative activity for each NTP is: ATP (1.00), CTP (0.80), GTP (0.67), and UTP (0.66). In contrast, *Ts*-Clp1 did not phosphorylate a single-stranded DNA (ssDNA) oligonucleotide, even with larger amounts of NTP (0.2–1.0 mM) (fig. 5*C*). However, *Ts*-Clp1 phosphorylated the ssDNA oligonucleotide when larger amount of the recombinant enzymes was used (>50 ng/reaction) (fig. 5*D*). These characteristics are quite similar to those of archaeal Clp1 (Jain and Shuman 2009). We also found that *Ts*-Clp1 phosphorylated both a double-stranded RNA (dsRNA) oligonucleotide and a dsRNA oligonucleotide containing a 3′ overhang in a similar manner to its phosphorylation of the ssRNA oligonucleotide (fig. 5*E* and *F*). As shown in figure 5*G*, the PNK activity of *Ts*-Clp1 was very heat stable. The enzyme even showed activity at 90 °C, and the preincubation of the reaction mixture at 90 °C before the enzyme was added did not affect the specific activity of *Ts*-Clp1.

## Discussion

We conducted a large-scale molecular evolutionary analysis of the Clp1 family proteins and systematically confirmed that this family of proteins is distributed throughout all three domains of life (table 2 and supplementary table S2, Supplementary Material online). Because our experimental data showed that bacterial *Ts*-Clp1 has PNK activity (fig. 5), we have also demonstrated that the Clp1 family proteins in all domains of life have associated PNK activity. In contrast, the number of species with a Clp1 family member differs across the three domains of life. All the representative eukaryote species examined had at least one Clp1 family protein, usually a Clp1 protein. Other Clp1 family proteins involved in prerRNA processing have evolved in a species-specific manner: Nol9 appears in the Metazoa, Plantae, and Amoebozoa, and Grc3 appears in the Fungi. These observations suggest that the ancestral *clp1* gene was duplicated in the common ancestor of the eukaryotes, and diversified functionally during their evolution (fig. 1). We also speculated that proteins in the “Others” category in table 2 may have similar functions to those of Nol9 or Grc3. However, further experimental analyses are required to determine whether the Clp1 proteins regulate both tRNA splicing and rRNA processing in the eukaryotic species that express only the Clp1 protein. In contrast, there are almost no duplicated *clp1* genes in either the Archaea or Bacteria. According to our analysis of complete genomes, the proportion of species with Clp1 family proteins was 85.8% in Eukarya, 41.4% in Archaea, and 0.9% in Bacteria (supplementary tables S3–S5, Supplementary Material online). Many archaeal tRNA genes contain a tRNA intron and exact pretRNA splicing is required to produce mature and functional tRNAs. However, approximately half the Archaea have no Clp1 enzyme. Therefore, it is suggested that the RNA ligase RtcB-dependent ligation pathway (the 3′-phosphate pathway) for tRNA exons is predominant in archaeal species without a *clp1* gene, which is required for the 5′-phosphate pathway (Englert et al. 2011; Yoshihisa 2014). However, we found a limited number of bacterial *clp1* genes in restricted and phylogenetically diverse bacterial species (fig. 2, table 2, supplementary tables S2 and S5, Supplementary Material online). Our research also strongly suggests that the bacterial proteins shown in table 2*B* are bacterial PNKs or bacterial Clp1 proteins, although they were initially annotated as GTPases or GTP-binding proteins based on the similarities of the Walker A and B motifs. Experimentally, *Ts*-Clp1 showed kinase activity at 90 °C, although the bacterium was isolated from an environment at 60 °C (Kieft et al. 1999; Gounder et al. 2011). *Ts*-Clp1 preferentially phosphorylates ssRNA oligonucleotides over ssDNA oligonucleotides, and its biochemical characteristics are very similar to those of the hyperthermophilic archaeal enzyme, *Ph*-Clp1 (Jain and Shuman 2009). Evolutionarily, the bacterial Clp1 proteins are distributed almost independently on the bacterial phylogenetic tree (fig. 2). On the basis of all these observations, we speculate that the bacterial *clp1* genes may have been acquired by horizontal gene transfer from one of the hyperthermophilic archaea. However, several features of bacterial Clp1 do not support this possibility, including its lack of the conserved C-terminal domain of archaeal Clp1. An alternative explanation is that a massive loss of *clp1* genes occurred in the early evolution of Bacteria, although the common ancestor of Bacteria had a *clp1* gene.

In terms of the Clp1 family protein structures, the PNK domain is highly conserved among all family members. Interestingly, there are also subfamily-specific domains. For example, eukaryotic Clp1 has conserved N-terminal (Clp1_eN) and C-terminal domains (Clp1_eC). Similarly, the Nol9 proteins, which are involved in prerRNA processing, have conserved N-terminal (Nol9_eN) and C-terminal domains (Nol9_eC) (supplementary figs. S5 and S6, Supplementary Material online). These findings suggest that the ancestral Clp1 and Nol9 proteins acquired their corresponding protein-specific N-terminal and C-terminal domains in the common ancestor of the eukaryotes in accordance with their specific substrates (pretRNA or prerRNA, respectively), resulting in their functional diversification during evolution (fig. 1). In the archaeal Clp1 proteins, only the C-terminal domain (Clp1_aC) is conserved (supplementary fig. S4, Supplementary Material online). There are no such conserved domains in Grc3. From an experimental perspective, both *Hs*-Clp1 and *Ce*-Clp1, which contain the eukaryotic Clp1_eN and Clp1_eC domains, mainly phosphorylate ssRNA under typical reaction conditions. It has also been reported that *Ce*-Clp1 very weakly phosphorylates ssDNA when incubated for a longer period (Weitzer and Martinez 2007; Dikfidan et al. 2014). In contrast, archaeal *Ph*-Clp1 does not contain the N-terminal domain (Jain and Shuman 2009) and bacterial *Ts*-Clp1 has neither the N- nor C-terminal domain. Using ssRNA as the substrate, we determined that the *Ts*-Clp1 *K*m value for ATP was 2.5 µM (supplementary fig. S10, Supplementary Material online). Prokaryotic *Ts*-Clp1 (*K*m, 2.5 µM) and *Ph*-Clp1 (*K*m, 16 µM) (Jain and Shuman 2009) phosphorylated the ssRNA at lower ATP concentrations than eukaryotic *Ce*-Clp1 (*K*m, 99 µM) (Dikfidan et al. 2014). However, these prokaryotic enzymes showed lower substrate specificity, also phosphorylating ssDNA. Therefore, we speculate that the N-terminal domain in eukaryotes may contribute the substrate specificity for RNA, but that the eukaryotic enzyme requires a relatively larger amount of ATP. On the basis of all these findings, the Clp1 enzymes can be broadly divided into two classes: Prokaryotic and eukaryotic. After the duplication of the primitive *clp1* gene, only the eukaryotic Clp1 protein acquired both the N-terminal and C-terminal domains, defining its RNA specificity. The N-terminal domain of *Ce*-Clp1 is required for its ATP-binding activity (Dikfidan et al. 2014). Although prokaryotic Clp1 lacks this N-terminal domain, its phosphorylation activity is intact. Therefore, it will be necessary to analyze the reaction mechanism at the structural level. There are two types of Clp1 orthologs: One with polynucleotide activity, such as human *Hs*-Clp1 (Weitzer and Martinez 2007), and the other without polynucleotide activity, such as yeast *Sc*-Clp1 (Ramirez et al. 2008). Although ATPase activity is not essential for mRNA 3′-processing, ATP binding may have a critical function in this event. Because *Sc*-Clp1 has no PNK activity, yeast tRNA ligase (Trl1) is instead responsible for the phosphorylation of the 3′ tRNA exon during pretRNA splicing. It has also been reported that *Hs*-Clp1 complemented a lethal kinase-defective Trl1 mutation in yeast (Ramirez et al. 2008). This result suggests that Clp1 and Trl1 share a functionally common regulatory mechanism in pretRNA splicing. We believe that the same is true of plant Rlg1 (Nagashima et al. 2016) and Clp1. However, the number of experimentally characterized enzymes is limited, and further research is required to properly understand the functions of these enzymes during evolution.

We also identified a set of large proteins containing the Clp1_P domain and other functional domains (fig. 3). As reported previously, Trl1 tRNA ligase contains three functional domains: Ligase, PNK, and CPDase domains (Wang and Shuman 2005; Englert et al. 2010). Although the PNK domain in Trl1 shows no significant similarity to that in Clp1, enzymes containing the PNK domain tend to have other functional domains. We speculate that the fundamental architecture of the PNK family proteins includes multiple functional domains. As far as we know, none of these large proteins has been characterized experimentally. Therefore, any analysis of large proteins containing the Clp1_P domain must also determine the functions of these other domains experimentally.

## Supplementary Material

Supplementary data are available at *Genome Biology and Evolution* online.

## Supplementary Material

evz195_Supplementary_Data

## References

[evz195-B1] Bernstein NK. 2009 Mechanism of DNA substrate recognition by the mammalian DNA repair enzyme, polynucleotide kinase. Nucleic Acids Res. 37(18):6161–6173. 19671525 10.1093/nar/gkp597 PMC2764422

[evz195-B2] Bernstein NK, et al 2005 The molecular architecture of the mammalian DNA repair enzyme, polynucleotide kinase. Mol Cell. 17(5):657–670. 15749016 10.1016/j.molcel.2005.02.012

[evz195-B3] Boratyn GM, et al 2013 BLAST: a more efficient report with usability improvements. Nucleic Acids Res. 41(W1):W29–33. 23609542 10.1093/nar/gkt282 PMC3692093

[evz195-B4] Braglia P, Heindl K, Schleiffer A, Martinez J, Proudfoot NJ. 2010 Role of the RNA/DNA kinase Grc3 in transcription termination by RNA polymerase I. EMBO Rep. 11(10):758–764. 20814424 10.1038/embor.2010.130 PMC2948184

[evz195-B5] Chaconas G, van de Sande JH. 1980 5′-32P labeling of RNA and DNA restriction fragments. Meth Enzymol. 65(1):75–85. 6154877 10.1016/s0076-6879(80)65012-5

[evz195-B6] Chalasani SL, et al 2018 Persistent 3′-phosphate termini and increased cytotoxicity of radiomimetic DNA double-strand breaks in cells lacking polynucleotide kinase/phosphatase despite presence of an alternative 3′-phosphatase. DNA Repair (Amst). 68:12–24. 29807321 10.1016/j.dnarep.2018.05.002 PMC6050096

[evz195-B7] Ciccarelli FD, et al 2006 Toward automatic reconstruction of a highly resolved tree of life. Science 311(5765):1283–1287. 16513982 10.1126/science.1123061

[evz195-B8] Cusick KD, et al 2016 Molecular mechanisms contributing to the growth and physiology of an extremophile cultured with dielectric heating. Appl Environ Microbiol. 82(20):6233–6246. 27520819 10.1128/AEM.02020-16 PMC5068158

[evz195-B9] Das U, Wang LK, Smith P, Shuman S. 2013 Structural and biochemical analysis of the phosphate donor specificity of the polynucleotide kinase component of the bacterial pnkp*hen1 RNA repair system. Biochemistry 52(27):4734–4743. 23721485 10.1021/bi400412x PMC3855621

[evz195-B10] de Vries H, et al 2000 Human pre-mRNA cleavage factor II(m) contains homologs of yeast proteins and bridges two other cleavage factors. EMBO J. 19(21):5895–5904. 11060040 10.1093/emboj/19.21.5895 PMC305781

[evz195-B11] Desai KK, Cheng CL, Bingman CA, Phillips GN Jr, Raines RT. 2014 A tRNA splicing operon: archease endows RtcB with dual GTP/ATP cofactor specificity and accelerates RNA ligation. Nucleic Acids Res. 42(6):3931–3942. 24435797 10.1093/nar/gkt1375 PMC3973293

[evz195-B12] Dikfidan A, et al 2014 RNA specificity and regulation of catalysis in the eukaryotic polynucleotide kinase Clp1. Mol Cell. 54(6):975–986. 24813946 10.1016/j.molcel.2014.04.005

[evz195-B13] Englert M, Sheppard K, Aslanian A, Yates JR 3rd, Soll D. 2011 Archaeal 3′-phosphate RNA splicing ligase characterization identifies the missing component in tRNA maturation. Proc Natl Acad Sci USA. 108(4):1290–1295. 21209330 10.1073/pnas.1018307108 PMC3029724

[evz195-B14] Englert M, Sheppard K, Gundllapalli S, Beier H, Soll D. 2010 *Branchiostoma floridae* has separate healing and sealing enzymes for 5′-phosphate RNA ligation. Proc Natl Acad Sci USA. 107(39):16834–16839. 20837552 10.1073/pnas.1011703107 PMC2947901

[evz195-B15] Finn RD, et al 2014 Pfam: the protein families database. Nucl Acids Res. 42(D1):D222–230. 24288371 10.1093/nar/gkt1223 PMC3965110

[evz195-B16] Fujishima K, et al 2009 Tri-split tRNA is a transfer RNA made from 3 transcripts that provides insight into the evolution of fragmented tRNAs in archaea. Proc Natl Acad Sci USA. 106(8):2683–2687. 19190180 10.1073/pnas.0808246106 PMC2650326

[evz195-B17] Fujishima K, Sugahara J, Tomita M, Kanai A. 2010 Large-scale tRNA intron transposition in the archaeal order Thermoproteales represents a novel mechanism of intron gain. Mol Biol Evol. 27(10):2233–2243. 20430862 10.1093/molbev/msq111

[evz195-B18] Gounder K, et al 2011 Sequence of the hyperplastic genome of the naturally competent *Thermus scotoductus* SA-01. BMC Genomics 12(1):577. 22115438 10.1186/1471-2164-12-577 PMC3235269

[evz195-B19] Gouy M, Guindon S, Gascuel O. 2010 SeaView version 4: a multiplatform graphical user interface for sequence alignment and phylogenetic tree building. Mol Biol Evol. 27(2):221–224. 19854763 10.1093/molbev/msp259

[evz195-B20] Gross S, Moore CL. 2001 Rna15 interaction with the A-rich yeast polyadenylation signal is an essential step in mRNA 3′-end formation. Mol Cell Biol. 21(23):8045–8055. 11689695 10.1128/MCB.21.23.8045-8055.2001 PMC99971

[evz195-B21] Hanada T, et al 2013 CLP1 links tRNA metabolism to progressive motor-neuron loss. Nature 495(7442):474–480. 23474986 10.1038/nature11923 PMC3674495

[evz195-B22] Heindl K, Martinez J. 2010 Nol9 is a novel polynucleotide 5′-kinase involved in ribosomal RNA processing. EMBO J 29(24):4161–4171. 21063389 10.1038/emboj.2010.275 PMC3018789

[evz195-B23] Henikoff S, Henikoff JG. 1992 Amino acid substitution matrices from protein blocks. Proc Natl Acad Sci USA. 89(22):10915–10919. 1438297 10.1073/pnas.89.22.10915 PMC50453

[evz195-B24] Holbein S, et al 2011 The P-loop domain of yeast Clp1 mediates interactions between CF IA and CPF factors in pre-mRNA 3′ end formation. PLoS One 6(12):e29139. 22216186 10.1371/journal.pone.0029139 PMC3245249

[evz195-B25] Jain R, Shuman S. 2009 Characterization of a thermostable archaeal polynucleotide kinase homologous to human Clp1. RNA 15(5):923–931. 19299550 10.1261/rna.1492809 PMC2673061

[evz195-B26] Kanai A, et al 2009 Characterization of a heat-stable enzyme possessing GTP-dependent RNA ligase activity from a hyperthermophilic archaeon, *Pyrococcus furiosus*. RNA 15(3):420–431. 19155324 10.1261/rna.1122109 PMC2657004

[evz195-B27] Katoh K, Standley DM. 2013 MAFFT multiple sequence alignment software version 7: improvements in performance and usability. Mol Biol Evol. 30(4):772–780. 23329690 10.1093/molbev/mst010 PMC3603318

[evz195-B28] Kieft TL, et al 1999 Dissimilatory reduction of Fe(III) and other electron acceptors by a *Thermus* isolate. Appl Environ Microbiol. 65(3):1214–1221. 10049886 10.1128/aem.65.3.1214-1221.1999 PMC91167

[evz195-B29] Leipe DD, Koonin EV, Aravind L. 2003 Evolution and classification of P-loop kinases and related proteins. J Mol Biol. 333(4):781–815. 14568537 10.1016/j.jmb.2003.08.040

[evz195-B30] Martins A, Shuman S. 2005 An end-healing enzyme from *Clostridium thermocellum* with 5′ kinase, 2′, 3′ phosphatase, and adenylyltransferase activities. RNA 11(8):1271–1280. 15987807 10.1261/rna.2690505 PMC1370810

[evz195-B31] Minvielle-Sebastia L, Preker PJ, Wiederkehr T, Strahm Y, Keller W. 1997 The major yeast poly(A)-binding protein is associated with cleavage factor IA and functions in premessenger RNA 3′-end formation. Proc Natl Acad Sci USA. 94(15):7897–7902. 9223284 10.1073/pnas.94.15.7897 PMC21526

[evz195-B32] Moore AD, Held A, Terrapon N, Weiner J, Bornberg-Bauer E. 2014 DoMosaics: software for domain arrangement visualization and domain-centric analysis of proteins. Bioinformatics 30(2):282–283. 24222210 10.1093/bioinformatics/btt640

[evz195-B33] Nagashima Y, Iwata Y, Mishiba K, Koizumi N. 2016 Arabidopsis tRNA ligase completes the cytoplasmic splicing of *bZIP60* mRNA in the unfolded protein response. Biochem Biophys Res Commun. 470(4):941–946. 26820526 10.1016/j.bbrc.2016.01.145

[evz195-B34] Noble CG, Beuth B, Taylor IA. 2006 Structure of a nucleotide-bound Clp1-Pcf11 polyadenylation factor. Nucleic Acids Res. 35(1):87–99. 17151076 10.1093/nar/gkl1010 PMC1761425

[evz195-B35] O'Leary NA. 2016 Reference sequence (RefSeq) database at NCBI: current status, taxonomic expansion, and functional annotation. Nucleic Acids Res. 44:D733–745. 26553804 10.1093/nar/gkv1189 PMC4702849

[evz195-B36] Orelle C, Dalmas O, Gros P, Di Pietro A, Jault JM. 2003 The conserved glutamate residue adjacent to the Walker-B motif is the catalytic base for ATP hydrolysis in the ATP-binding cassette transporter BmrA. J Biol Chem. 278(47):47002–47008. 12968023 10.1074/jbc.M308268200

[evz195-B37] Paushkin SV, Patel M, Furia BS, Peltz SW, Trotta CR. 2004 Identification of a human endonuclease complex reveals a link between tRNA splicing and pre-mRNA 3′ end formation. Cell 117(3):311–321. 15109492 10.1016/s0092-8674(04)00342-3

[evz195-B38] Phizicky EM, Schwartz RC, Abelson J. 1986 *Saccharomyces cerevisiae* tRNA ligase. Purification of the protein and isolation of the structural gene. J Biol Chem. 261(6):2978–2986. 3512545

[evz195-B39] Pillon MC, Sobhany M, Stanley RE. 2018 Characterization of the molecular crosstalk within the essential Grc3/Las1 pre-rRNA processing complex. RNA 24(5):721–738. 29440475 10.1261/rna.065037.117 PMC5900568

[evz195-B40] Popow J, Jurkin J, Schleiffer A, Martinez J. 2014 Analysis of orthologous groups reveals archease and DDX1 as tRNA splicing factors. Nature 511(7507):104–107. 24870230 10.1038/nature13284 PMC4417724

[evz195-B41] Potter SC, et al 2018 HMMER web server: 2018 update. Nucleic Acids Res. 46(W1):W200–W204. 29905871 10.1093/nar/gky448 PMC6030962

[evz195-B42] Ramirez A, Shuman S, Schwer B. 2008 Human RNA 5′-kinase (hClp1) can function as a tRNA splicing enzyme in vivo. RNA 14(9):1737–1745. 18648070 10.1261/rna.1142908 PMC2525948

[evz195-B43] Ruegsegger U, Leber JH, Walter P. 2001 Block of *HAC1* mRNA translation by long-range base pairing is released by cytoplasmic splicing upon induction of the unfolded protein response. Cell 107:103–114. 11595189 10.1016/s0092-8674(01)00505-0

[evz195-B44] Sato A, et al 2011 GTP-dependent RNA 3′-terminal phosphate cyclase from the hyperthermophilic archaeon *Pyrococcus furiosus*. Genes Cells 16(12):1190–1199. 22074260 10.1111/j.1365-2443.2011.01561.x

[evz195-B45] Schwer B, Sawaya R, Ho CK, Shuman S. 2004 Portability and fidelity of RNA-repair systems. Proc Natl Acad Sci USA. 101(9):2788–2793. 14973195 10.1073/pnas.0305859101 PMC365698

[evz195-B46] Shuman S, Hurwitz J. 1979 5′-Hydroxyl polyribonucleotide kinase from HeLa cell nuclei. Purification and properties. J Biol Chem. 254(20):10396–10404. 226541

[evz195-B47] Stamatakis A. 2014 RAxML version 8: a tool for phylogenetic analysis and post-analysis of large phylogenies. Bioinformatics 30(9):1312–1313. 24451623 10.1093/bioinformatics/btu033 PMC3998144

[evz195-B48] Sugahara J, et al 2008 Comprehensive analysis of archaeal tRNA genes reveals rapid increase of tRNA introns in the order thermoproteales. Mol Biol Evol. 25(12):2709–2716. 18832079 10.1093/molbev/msn216

[evz195-B49] Tahbaz N, Subedi S, Weinfeld M. 2012 Role of polynucleotide kinase/phosphatase in mitochondrial DNA repair. Nucleic Acids Res. 40(8):3484–3495. 22210862 10.1093/nar/gkr1245 PMC3333865

[evz195-B50] The UniProt Consortium 2017 UniProt: the universal protein knowledgebase. Nucleic Acids Res. 45:D158–D169. 27899622 10.1093/nar/gkw1099 PMC5210571

[evz195-B51] Wang LK, Das U, Smith P, Shuman S. 2012 Structure and mechanism of the polynucleotide kinase component of the bacterial Pnkp-Hen1 RNA repair system. RNA 18(12):2277–2286. 23118415 10.1261/rna.036061.112 PMC3504678

[evz195-B52] Wang LK, Lima CD, Shuman S. 2002 Structure and mechanism of T4 polynucleotide kinase: an RNA repair enzyme. EMBO J. 21(14):3873–3880. 12110598 10.1093/emboj/cdf397 PMC126130

[evz195-B53] Wang LK, Schwer B, Englert M, Beier H, Shuman S. 2006 Structure–function analysis of the kinase-CPD domain of yeast tRNA ligase (Trl1) and requirements for complementation of tRNA splicing by a plant Trl1 homolog. Nucleic Acids Res. 34(2):517–527. 16428247 10.1093/nar/gkj441 PMC1345694

[evz195-B54] Wang LK, Shuman S. 2005 Structure–function analysis of yeast tRNA ligase. RNA 11(6):966–975. 15923379 10.1261/rna.2170305 PMC1370781

[evz195-B55] Waterhouse AM, Procter JB, Martin DM, Clamp M, Barton GJ. 2009 Jalview Version 2 – a multiple sequence alignment editor and analysis workbench. Bioinformatics 25(9):1189–1191. 19151095 10.1093/bioinformatics/btp033 PMC2672624

[evz195-B56] Weinfeld M, Mani RS, Abdou I, Aceytuno RD, Glover JN. 2011 Tidying up loose ends: the role of polynucleotide kinase/phosphatase in DNA strand break repair. Trends Biochem Sci. 36(5):262–271. 21353781 10.1016/j.tibs.2011.01.006 PMC3134258

[evz195-B57] Weitzer S, Hanada T, Penninger JM, Martinez J. 2015 CLP1 as a novel player in linking tRNA splicing to neurodegenerative disorders. Wires RNA 6(1):47–63. 25142875 10.1002/wrna.1255

[evz195-B58] Weitzer S, Martinez J. 2007 The human RNA kinase hClp1 is active on 3′ transfer RNA exons and short interfering RNAs. Nature 447(7141):222–226. 17495927 10.1038/nature05777

[evz195-B59] Wu M, Eisen JA. 2008 A simple, fast, and accurate method of phylogenomic inference. Genome Biol. 9(10):R151. 18851752 10.1186/gb-2008-9-10-r151 PMC2760878

[evz195-B60] Xing D, Zhao H, Li QQ. 2008 Arabidopsis CLP1-SIMILAR PROTEIN3, an ortholog of human polyadenylation factor CLP1, functions in gametophyte, embryo, and postembryonic development. Plant Physiol. 148(4):2059–2069. 18971429 10.1104/pp.108.129817 PMC2593670

[evz195-B61] Yoshihisa T. 2014 Handling tRNA introns, archaeal way and eukaryotic way. Front Genet. 5:213. 25071838 10.3389/fgene.2014.00213 PMC4090602

[evz195-B62] Zhang C, Chan CM, Wang P, Huang RH. 2012 Probing the substrate specificity of the bacterial Pnkp/Hen1 RNA repair system using synthetic RNAs. RNA 18(2):335–344. 22190744 10.1261/rna.030502.111 PMC3264919

[evz195-B63] Zhu H, Smith P, Wang LK, Shuman S. 2007 Structure–function analysis of the 3′ phosphatase component of T4 polynucleotide kinase/phosphatase. Virology 366(1):126–136. 17493655 10.1016/j.virol.2007.03.059 PMC2761019

