# Supplementary material for: Large-Scale Molecular Evolutionary Analysis Uncovers a Variety of Polynucleotide Kinase Clp1 Family Proteins in the Three Domains of Life
Source: Genome Biol Evol. 2019 Sep 12;11(10):2713–26. doi: 10.1093/gbe/evz195 (PMC6777427; doi:10.1093/gbe/evz195)
Supplement: evz195_Supplementary_Data [file evz195_supplementary_data.zip › Supplementary Information_R.pdf]

Supplementary Information

Large-scale Molecular Evolutionary Analysis Uncovers a Variety of Polynucleotide Kinase Clp1 Family Proteins in the Three Domains of Life

Motofumi Saito[1,2], Asako Sato[1], Shohei Nagata[1,2], Satoshi Tamaki[1],
Masaru Tomita[1,2,3], Haruo Suzuki[1,3], and Akio Kanai[1,2,3] *

[1]Institute for Advanced Biosciences, Keio University, Tsuruoka 997-0017, Japan
[2]Systems Biology Program, Graduate School of Media and Governance, Keio University, Fujisawa 252-0882, Japan
[3]Faculty of Environment and Information Studies, Keio University, Fujisawa 252-0882, Japan

*Corresponding Author

Akio Kanai, PhD
Institute for Advanced Biosciences, Keio University
Tsuruoka, Yamagata 997-0017, Japan
Tel: +81-235-29-0524
Fax: +81-235-29-0525
E-mail: akio@sfc.keio.ac.jp

Supplementary Table S1. Summary of Clp1 query sequences used in this study

Protein name	UniProt AC	Polynucleotide kinase region	Total aa length	Species	Domain
Polyribonucleotide 5'-hydroxyl-kinase Clp1	Q92989	121-307	425	*Homo sapiens*	Eukarya
Polynucleotide 5'-hydroxyl-kinase Nol9	Q5SY16	306-471	702	*Homo sapiens*	Eukarya
mRNA cleavage and polyadenylation factor Clp1	Q08685	130-335	445	*Saccharomyces cerevisiae*	Eukarya
Polynucleotide 5'-hydroxyl-kinase Grc3	Q07845	246-450	632	*Saccharomyces cerevisiae*	Eukarya
Polyribonucleotide 5'-hydroxyl-kinase PH0197	O57936	43-217	361	*Pyrococcus horikoshii*	Archaea
GTPase or GTP-binding protein-like protein	A5G778	41-216	295	*Geobacter uraniireducens*	Bacteria

Supplementary Table S2. List of species containing Clp1 family proteins

	Class (Group)	Number of species with Clp1	Number of Clp1 family proteins
**Eukarya**	Opisthokonta	1,180	2,516
	Viridiplantae	138	414
	Stramenopiles	35	90
	Euglenozoa	25	51
	Alveolata	19	27
	Amoebozoa	15	21
	Rhodophyta	4	7
	Rhizaria	3	5
	Parabasalia	2	6
	Haptophyceae	2	2
	Heterolobosea	1	2
	Cryptophyta	1	1
	Apusozoa	1	2
	Total	1,426	3,144
**Archaea**	TACK group	106	127
	Euryarchaeota	94	96
	Unclassified Archaea	10	13
	Environmental samples	1	2
	Total	211	238
**Bacteria**	Unclassified Bacteria	42	50
	Proteobacteria	39	41
	Terrabacteria group	19	21
	PVC group	19	20
	FCB group	6	6
	Nitrospirae	6	7
	Nitrospinae/Tectomicrobia	4	4
	Dictyoglomi	4	4
	Thermodesulfobacteria	1	1
	Spirochaetes	1	1
	Calditrichaeota	1	1
	Aquificae	1	1
	Environmental samples	1	1
	Total	144	158
	Total	1,781	3,540

Supplementary Table S3. Proportion of species in Eukarya containing Clp1 family proteins (at the phylum and kingdom levels).

Class (Group)		Clp1	Nol9	Grc3	Others	Number of Clp1 family protein	Number of species with Clp1 family protein	Number of species registered	Ratio (%)
Alveolata	Apicomplexa	2	1	0	6	9	7	27	25.9
	Ciliophora	0	0	0	0	0	0	1	0.0
Amoebozoa	Mycetozoa	0	0	0	2	2	1	2	50.0
Cryptophyta	Cryptomonadales	0	0	0	0	0	0	2	0.0
	Pyrenomonadales	0	0	0	0	0	0	1	0.0
Euglenozoa	Kinetoplastida	8	0	0	8	16	8	8	100.0
Fungi	Dikarya	11	0	13	79	103	53	58	91.4
	Microsporidia	0	0	0	4	4	4	4	100.0
Metazoa	Chordata	83	85	0	202	370	85	87	97.7
	Ecdysozoa	13	13	0	29	55	20	22	90.9
	Lophotrochozoa	3	3	0	4	10	3	3	100.0
	Platyhelminthes	1	0	0	1	2	1	1	100.0
Rhizaria	Cercozoa	0	0	0	0	0	0	1	0.0
Rhodophyta	Bangiophyceae	1	0	0	0	1	1	1	100.0
Stramenopiles	Bacillariophyta	0	0	0	3	3	2	2	100.0
Viridiplantae	Chlorophyta	0	0	0	6	6	4	4	100.0
	Streptophyta	56	54	0	162	272	58	64	90.6
	Total	178	156	13	506	853	247	288	85.8

Supplementary Table S4. Species of Eukarya containing Clp1 family proteins.

This Table is supplied as a separate Excel file.

Supplementary Table S5. Proportion of species containing Clp1 family proteins in prokaryotes

	Class (Group)	Number of species registered	Number of Clp1 family protein	Ratio(%)
**Archaea**	Methanomicrobia	29	3	10.3
	Halobacteria	26	0	0.0
	Thermococci	24	24	100.0
	Thermoprotei	19	19	100.0
	Methanobacteria	13	0	0.0
	Methanococci	12	4	33.3
	Archaeoglobi	7	7	100.0
	Thermoplasmata	7	0	0.0
	Nitrososphaeria	1	1	100.0
	Archaeon	1	0	0.0
	DHVE2	1	0	0.0
	Total	140	58	41.4
**Bacteria**	Proteobacteria	658	6	0.9
	Firmicutes	293	1	0.3
	Actinobacteria	244	0	0.0
	Bacteroidetes	99	1	1.0
	Tenericutes	52	0	0.0
	Spirochaetes	33	0	0.0
	Cyanobacteria	29	0	0.0
	Deinococcus-Thermus	18	2	11.1
	Thermotogae	16	0	0.0
	Chlamydiae	12	0	0.0
	Chlorobi	11	0	0.0
	Chloroflexi	11	0	0.0
	Planctomycetes	8	0	0.0
	Aquificae	8	1	12.5
	Verrucomicrobia	7	0	0.0
	Fusobacteria	6	0	0.0
	Acidobacteria	6	0	0.0
	Nitrospirae	5	0	0.0
	Deferribacteres	4	0	0.0
	Synergistetes	4	0	0.0
	Thermodesulfobacteria	4	1	25.0
	Elusimicrobia	3	0	0.0
	Gemmatimonadetes	2	0	0.0
	Ignavibacteriae	2	0	0.0
	Thermobaculum	1	0	0.0
	Kiritimatiellaeota	1	0	0.0
	Fibrobacteres	1	0	0.0
	Dictyoglomi	1	1	100.0
	Chrysiogenetes	1	0	0.0
	Calditrichaeota	1	1	100.0
	Caldiserica	1	0	0.0
	Armatimonadetes	1	0	0.0
	Total	1,543	14	0.9
	Total	1,683	72	4.3

Supplementary Table S6 List of large proteins containing Clp1 polynucleotide kinase domain(s)

aa length	UniProt AC	Species	Protein annotation
1007	A0A2H9TH54	*Paramicrosporidium saccamoebae*	Uncharacterized protein
1007	C3Z8N7	*Branchiostoma floridae*	Protein CLP1 homolog
1009	A0A146F5J7	*Aspergillus luchuensis*	mRNA cleavage and polyadenylation factor CLP1
1015	A0A084W2E3	*Anopheles sinensis*	Uncharacterized protein
1021	A0A139WEM6	*Tribolium castaneum*	Protein CLP1 homolog
1023	B3NPX7	*Drosophila erecta*	Uncharacterized protein
1026	A0A261BI83	*Caenorhabditis latens*	Uncharacterized protein (Fragment)
1032	B4P6R6	*Drosophila yakuba*	Uncharacterized protein
1034	A0A1G4IIH9	*Trypanosoma equiperdum*	Molybdopterin guanine dinucleotide synthesis protein B, putative
1034	A0A1S4FYI6	*Aedes aegypti*	Uncharacterized protein
1034	A0A2G7G0R6	*Aspergillus arachidicola*	mRNA cleavage and polyadenylation factor CLP1
1034	G0UR73	*Trypanosoma congolense*	Uncharacterized protein
1034	Q57VM9	*Trypanosoma brucei brucei*	Uncharacterized protein
1035	A0A182H4R7	*Aedes albopictus*	Uncharacterized protein
1037	A0A1A9UCT0	*Glossina austeni*	Uncharacterized protein
1037	C9ZUP2	*Trypanosoma brucei gambiense*	Uncharacterized protein
1038	A0A182GS53	*Aedes albopictus*	Uncharacterized protein
1041	Q16K19	*Aedes aegypti*	AAEL013142-PA
1043	A0A0V1H769	*Trichinella zimbabwensis*	Protein CLP1-like protein (Fragment)
1043	A0A182XJD2	*Anopheles quadriannulatus*	Uncharacterized protein
1043	A0A2U3EC60	*Purpureocillium lilacinum*	Grc3
1046	A0A100INU4	*Aspergillus niger*	mRNA cleavage and polyadenylation factor CLP1
1048	A0A0V1IFH5	*Trichinella pseudospiralis*	Protein CLP1-like protein
1048	A0A0V1LSZ1	*Trichinella nativa*	Protein CLP1 homolog
1054	A0A0E9NA66	*Saitoella complicata* NRRL Y-17804	Uncharacterized protein
1054	A0A0F7SU13	*Phaffia rhodozyma*	Polynucleotide 5-hydroxyl-kinase grc3
1054	A0A0J9RE02	*Drosophila simulans*	Uncharacterized protein
1054	A0A0V1BKN3	*Trichinella spiralis*	Protein CLP1 homolog
1056	A0A0V1LSR2	*Trichinella nativa*	Protein CLP1 homolog
1056	E5S487	*Trichinella spiralis*	Protein CLP1 homolog
1058	A0A0V1MCA0	*Trichinella papuae*	Polynucleotide 5'-hydroxyl-kinase Nol9
1060	A0A0V0S0U0	*Trichinella nelsoni*	Protein CLP1 homolog
1060	A0A0V1DEW9	*Trichinella britovi*	Protein CLP1 homolog
1060	A0A0V1IGN0	*Trichinella pseudospiralis*	Protein CLP1 homolog
1060	A0A0V1M874	*Trichinella papuae*	Protein CLP1 homolog
1060	A0A0V1PG76	*Trichinella sp. T8*	Protein CLP1 homolog
1060	A0A1Y3EBI8	*Trichinella nativa*	Protein CLP1 homolog
1060	W8AWV5	*Ceratitis capitata*	Polynucleotide 5'-hydroxyl-kinase Nol9
1061	A0A1X6MWR6	*Postia placenta* MAD-698-R-SB12	Uncharacterized protein
1061	B8P402	*Postia placenta*	Predicted protein
1062	B3MF52	*Drosophila ananassae*	Uncharacterized protein
1065	A0A182KAI0	*Anopheles christyi*	Uncharacterized protein
1065	Q7PPX9	*Anopheles gambiae*	AGAP005161-PA
1067	A0A094A357	*Pseudogymnoascus sp.* VKM F-3557	Uncharacterized protein (Fragment)
1067	A0A0V0XSX5	*Trichinella pseudospiralis*	Protein CLP1 homolog
1068	K1W7Y0	*Trichosporon asahii var. asahii*	Uncharacterized protein
1069	A0A1X0NQ27	*Trypanosoma theileri*	Uncharacterized protein
1070	A0A0V1M8R6	*Trichinella papuae*	Protein CLP1 homolog
1074	A0A0V1M9J7	*Trichinella papuae*	Protein CLP1 homolog
1077	A0A0V0TZM9	*Trichinella murrelli*	Protein CLP1 homolog
1083	A0A094FIZ3	*Pseudogymnoascus sp.* VKM F-4518	mRNA cleavage and polyadenylation factor CLP1
1083	A0A0V1IEZ2	*Trichinella pseudospiralis*	Protein CLP1 homolog
1083	E3M674	*Caenorhabditis remanei*	Uncharacterized protein
1087	A0A0V1DYP0	*Trichinella pseudospiralis*	Protein CLP1-like protein (Fragment)
1089	A0A0V0WB90	*Trichinella sp.* T6	Protein CLP1-like protein (Fragment)
1095	A0A0V1H6A5	*Trichinella zimbabwensis*	Protein CLP1-like protein (Fragment)
1095	A0A1Q3EYP7	*Culex tarsalis*	Putative mrna cleavage and polyadenylation factor clp1 p-loop
1095	A0A1Q3EYR0	*Culex tarsalis*	Putative mrna cleavage and polyadenylation factor clp1 p-loop
1096	A0A1W4VC11	*Drosophila ficusphila*	polynucleotide 5'-hydroxyl-kinase Nol9 isoform X2
1098	A0A1Q3EYT6	*Culex tarsalis*	Putative mrna cleavage and polyadenylation factor clp1 p-loop
1099	A0A0V1H6P4	*Trichinella zimbabwensis*	Protein CLP1-like protein (Fragment)

aa length	UniProt AC	Species	Protein annotation
1099	A0A1R1YAU1	*Smittium culicis*	Polynucleotide 5'-hydroxyl-kinase Nol9
1100	A0A1R1XNP2	*Smittium culicis*	Polynucleotide 5'-hydroxyl-kinase Nol9
1102	A0A1B0BE37	*Glossina palpalis gambiensis*	Uncharacterized protein
1107	A0A0V1DY04	*Trichinella pseudospiralis*	Protein CLP1-like protein (Fragment)
1107	A0A0V1FUM1	*Trichinella pseudospiralis*	Protein CLP1 homolog
1113	A0A094CVB1	*Pseudogymnoascus sp.* VKM F-4516	Uncharacterized protein
1114	A0A094E4W2	*Pseudogymnoascus sp.* VKM F-4513	Uncharacterized protein
1115	A0A1W4VBL5	*Drosophila ficusphila*	polynucleotide 5'-hydroxyl-kinase Nol9 isoform X1
1123	V9DDX3	*Cladophialophora carrionii* CBS 160.54	Uncharacterized protein
1126	A0A0V1DY01	*Trichinella pseudospiralis*	Protein CLP1-like protein (Fragment)
1126	A0A1A9Z821	*Glossina pallidipes*	Uncharacterized protein
1130	A0A2A6CBA4	*Pristionchus pacificus*	Pik-1
1138	W3VK17	*Pseudozyma aphidis*	mRNA cleavage and polyadenylation factor CLP1
1141	S8CML7	*Genlisea aurea*	Uncharacterized protein
1169	A0A0K8UR84	*Bactrocera latifrons*	Polynucleotide 5'-hydroxyl-kinase Nol9
1176	A0A034V6T5	*Bactrocera dorsalis*	Polynucleotide 5'-hydroxyl-kinase Nol9
1180	A0A093X5P9	*Pseudogymnoascus sp.* VKM F-3808	Uncharacterized protein
1191	A0A1A9WEH5	*Glossina brevipalpis*	Uncharacterized protein
1193	A0A1A9Y4S8	*Glossina fuscipes fuscipes*	Uncharacterized protein
1196	Q4QIN4	*Leishmania major*	Uncharacterized protein
1199	A0A1B0FQQ2	*Glossina morsitans morsitans*	Uncharacterized protein
1201	A0A088RIM6	*Leishmania panamensis*	Uncharacterized protein
1201	E9AL70	*Leishmania mexicana*	Uncharacterized protein
1203	A0A1J1H8X2	*Plasmodium relictum*	Uncharacterized protein
1235	A0A2P8YI61	*Blattella germanica*	Cullin-2
1246	A0A061H633	*Anthracocystis flocculosa* PF-1	Uncharacterized protein
1248	A0A232FGH9	*Trichomalopsis sarcophagae*	Uncharacterized protein (Fragment)
1259	A0A388KMW0	*Chara braunii*	Uncharacterized protein
1268	A0A0A1XDF1	*Zeugodacus cucurbitae*	Polynucleotide 5'-hydroxyl-kinase Nol9
1302	A0A1I8NTU5	*Stomoxys calcitrans*	Uncharacterized protein
1303	A0A2H6KFQ7	*Babesia ovata*	Polynucleotide 5-hydroxyl-kinase Nol9
1312	A0A0N1IIH1	*Leptomonas seymouri*	Uncharacterized protein
1345	A4H508	*Leishmania braziliensis*	Uncharacterized protein
1368	A4HT70	*Leishmania infantum*	Molybdopterin
1370	E9B956	*Leishmania donovani*	Uncharacterized protein
1397	A0A0M9FR28	*Leptomonas pyrrhocoris*	Uncharacterized protein
1399	A0A1V9Y874	*Thraustotheca clavata*	Uncharacterized protein
1416	A0A1V9Z9K0	*Achlya hypogyna*	Uncharacterized protein
1430	A0A075ANU2	*Rozella allomycis* CSF55	14-3-3 protein domain-containing protein
1447	A0A178U9P2	*Arabidopsis thaliana*	Translation factor GUF1 homolog, mitochondrial
1448	A0A0L0CDM8	*Lucilia cuprina*	Polynucleotide 5'-hydroxyl-kinase Nol9
1452	A0A1I8N5Z6	*Musca domestica*	Uncharacterized protein
1471	A0A1B8AJI7	*Fusarium poae*	mRNA cleavage and polyadenylation factor CLP1
1552	A0A0K6FVA0	*Rhizoctonia solani*	Fanconi-associated nuclease
1624	A0A146HIK0	*Mycena chlorophos*	Uncharacterized protein
1631	A0A0B2WWM9	*Metarhizium album*	mRNA cleavage and polyadenylation factor CLP1
1636	A0A367L4Z2	*phiocordyceps polyrhachis-furcata* BCC 54312	Uncharacterized protein (Fragment)
1687	A0A081CP05	*Pseudozyma antarctica*	mRNA cleavage and polyadenylation factor CLP1
1772	A0A0G4N8C9	*Verticillium longisporum*	mRNA cleavage and polyadenylation factor CLP1
1865	A0A063BMC7	*Ustilaginoidea virens*	mRNA cleavage and polyadenylation factor CLP1
1920	A0A1Q9CS63	*Symbiodinium microadriaticum*	Polynucleotide 5'-hydroxyl-kinase Grc3
2152	A0A1J8QET8	*Rhizopogon vesiculosus*	Uncharacterized protein
2385	A0A0V1HGY2	*Trichinella zimbabwensis*	Voltage-dependent calcium channel unc-36
2405	A0A0V1HG97	*Trichinella zimbabwensis*	Voltage-dependent calcium channel unc-36
2567	A0A0V1HGN9	*Trichinella zimbabwensis*	Voltage-dependent calcium channel unc-36
2613	A0A0V1HIU3	*Trichinella zimbabwensis*	Voltage-dependent calcium channel unc-36
2624	A0A0V1HG31	*Trichinella zimbabwensis*	Voltage-dependent calcium channel unc-36
2652	A0A0V1HHG0	*Trichinella zimbabwensis*	Voltage-dependent calcium channel unc-36
2666	A0A0V1HG96	*Trichinella zimbabwensis*	Voltage-dependent calcium channel unc-36
2670	A0A0V1HGJ2	*Trichinella zimbabwensis*	Voltage-dependent calcium channel unc-36
2728	F0YEV4	*Aureococcus anophagefferens*	Uncharacterized protein

Supplementary Table S7. Summary of bacterial Clp1 proteins predicted in this study

UniProt AC	Organism name	aa length
A0A1J0LT23	*Thermus brockianus*	233
E8PQM6	*Thermus scotoductus*	240
A0A0S3QVY2	*Thermosulfidibacter takaii*	272
B8II67	*Methylobacterium nodulans*	276
F3Z1E4	*Desulfovibrio africanus*	286
B5YBE4	*Dictyoglomus thermophilum*	287
B2IH37	*Beijerinckia indica subsp. indica*	288
H1XND2	*Caldithrix abyssi*	294
D5BZ19	*Nitrosococcus halophilus*	294
A5G778	*Geobacter uraniireducens*	281
D0MF96	*Rhodothermus marinus*	310
WP_083817645*	*Thermodesulfatator indicus*	362
C9RCZ7	*Ammonifex degensii*	368
F2NCI2	*Desulfobacca acetoxidans*	374
Q8U4H6	*Pyrococcus furiosus*	354
O57936	*Pyrococcus horikoshii*	361

*GenBank ID

Supplementary Table S8. Oligonucleotide sequences used for the phosphorylation assay

Oligonucleotide name	DNA/RNA	Oligonucleotide Sequence	Length (nt)
R20-FAM	RNA	5' - UAAUACGACUCACUAUAGGG -3' (FAM)	20
R20-comp	RNA	5' - CCCUAUAGUGAGUCGUAUUA -3'	20
R22-comp-oh	RNA	5' - CCCUAUAGUGAGUCGUAUUA**GC** -3'	22
D20-FAM	DNA	5' - TAATACGACTCACTATAGGG -3' (FAM)	20

Both oligonucleotides R20-comp and R22-comp-oh have sequences complementary to that of the R20-FAM oligonucleotide. This is also true for the D20-FAM oligonucleotides. FAM, fluorescein amidite; oh, overhang sequence (underlined).

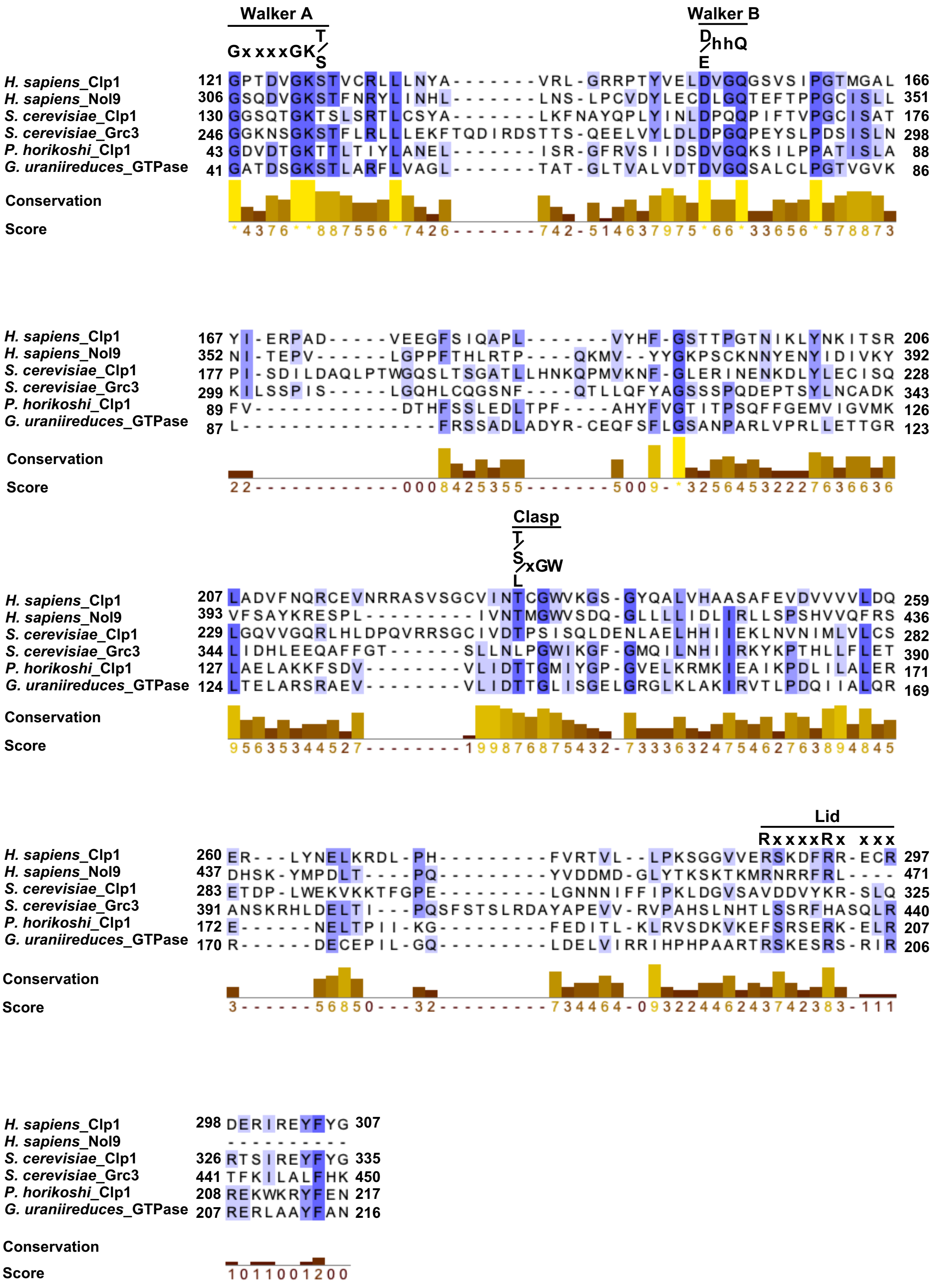

Supplementary Figure S1. Alignment of six polynucleotide kinase domain regions of representative Clp1 or Clp1-related proteins used as queries in this study. Amino acid sequences were aligned with MAFFT and the alignment was visualized with Jalview. Identical amino acid residues are indicated in blue and partly conserved amino acid residues are indicated in light blue. Gaps (–) were inserted to maximize the number of amino acid matches. The conservation score for each amino acid position is indicated as one of 12 ranks (0–11). Identical amino acids (rank 11) are indicated with asterisks (Livingstone and Barton 1993). See Supplementary Table S1 and Figure 4 for detailed information including UniProt accession number (AC) for each protein and species and for the conserved motifs, respectively.

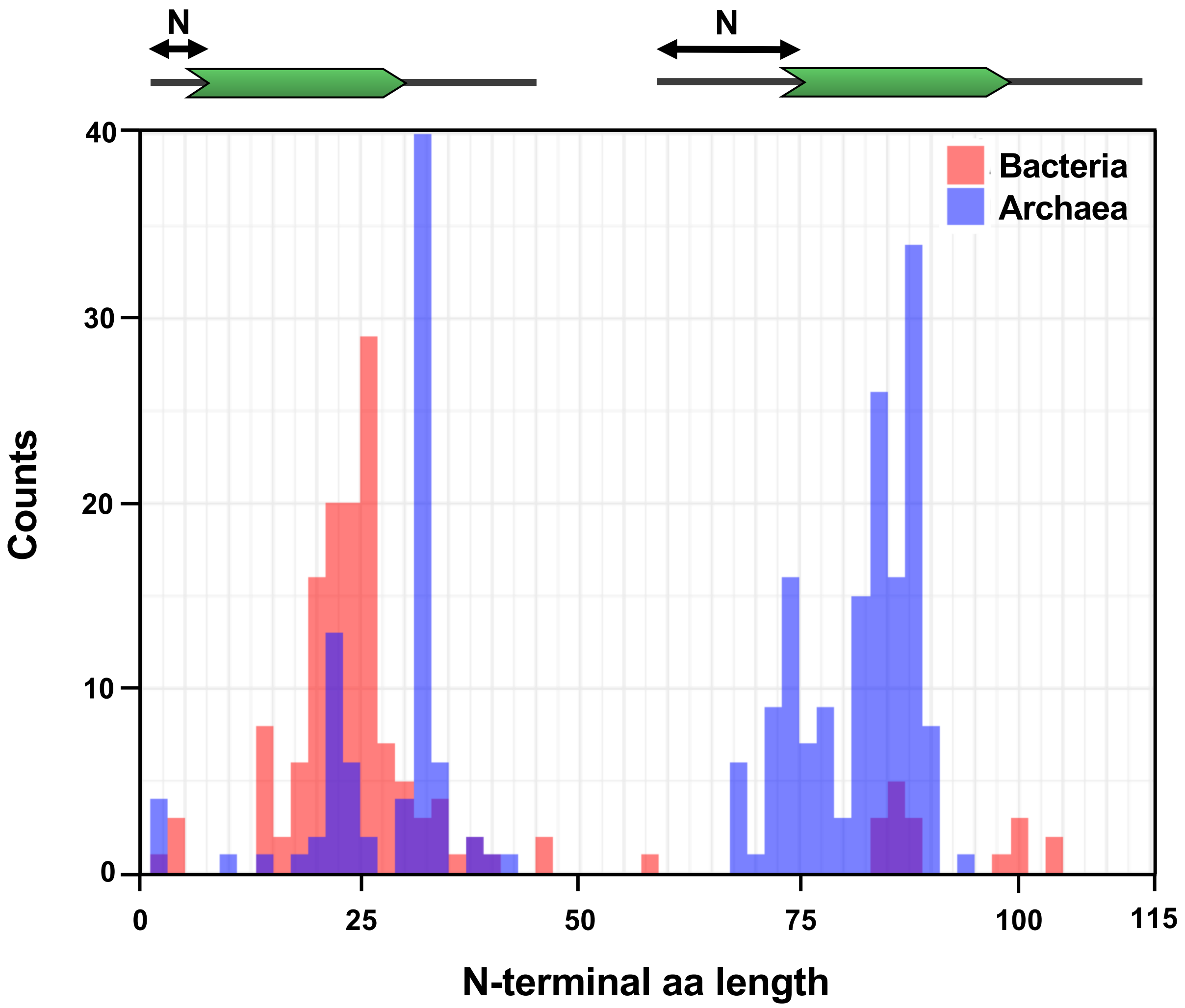

Supplementary Figure S2. Distribution of N-terminal lengths of prokaryotic Clp1 family proteins. 235 archaeal and 149 bacterial Clp1 family proteins obtained from UniProtKB were used for the analysis. The vertical axis indicates the number of proteins and the horizontal axis represents the N-terminal amino acid length. Here, 'N-terminal' is defined as the region from the first methionine to the amino acid located just before the polynucleotide kinase domain. Schematic representation of the shorter and longer N-terminal domains is shown on the top, in which the polynucleotide kinase domain is shown in green. The size distribution of N-terminal lengths had two peaks corresponding to the short and long forms. The short peak mainly consisted of bacterial Clp1 proteins (red) and the long peak mainly consisted of archaeal Clp1 proteins (blue).

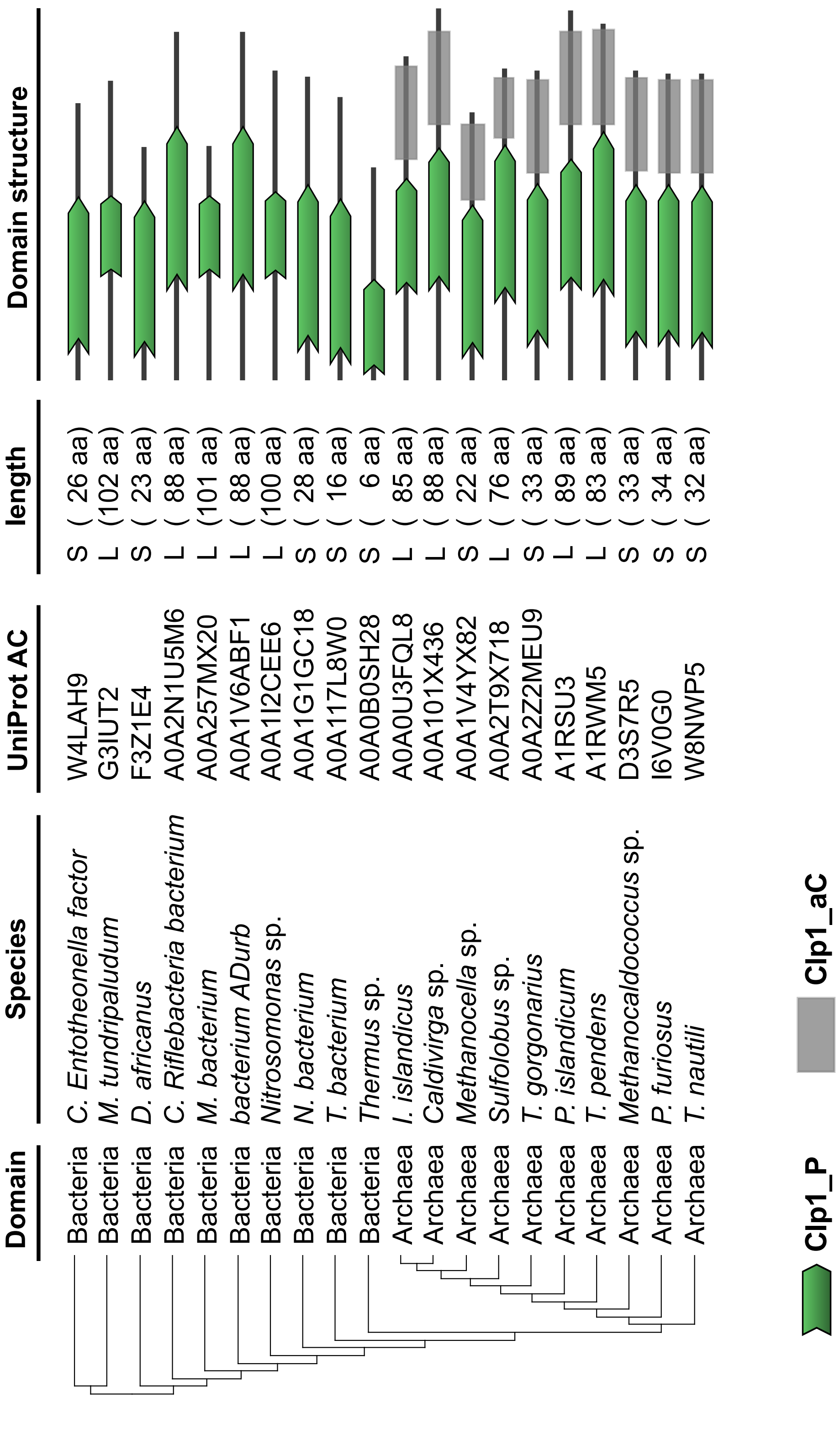

Supplementary Figure S3. Phylogeny and domain structure of prokaryotic Clp1 family proteins. Phylogenetic tree was constructed based on the amino acid sequences of 20 selected Clp1 family proteins with the neighbor-joining method. N-terminal lengths are classified into two groups: short (S), < 60 aa, and long (L), > 61 aa. See also Supplementary Figure S2. Domains were visualized with DoMosaics. Domains are defined as follows: Clp1_P, polynucleotide kinase domain; Clp1_aC, Clp1 C-terminal domain in archaea. The organisms are: *Candidatus Entotheonella factor*, *Methylobacter tundripaludum*, *Desulfovibrio africanus* str. Walvis Bay, *Candidatus Riflebacteria* bacterium HGW-*Riflebacteria-2*, Methylococcaceae bacterium NSP1-1, bacterium ADurb.Bin157, *Nitrosomonas* sp. Nm166, Nitrospirae bacterium GWD2_57_9, Thermoanaerobacterales bacterium 50_218, *Thermus* sp. 2.9, *Ignicoccus islandicus* DSM 13165, *Caldivirga* sp. MG_3, *Methanocella* sp. PtaU1.Bin125, *Sulfolobus* sp. SCGC AB-777_G06, *Thermococcus gorgonarius*, *Pyrobaculum islandicum* (strain DSM 4184/JCM 9189/GEO3), *Thermofilum pendens* (strain DSM 2475/Hrk 5), *Methanocaldococcus* sp. (strain FS406-22), *Pyrococcus furiosus* COM1, and *Thermococcus nautili*.

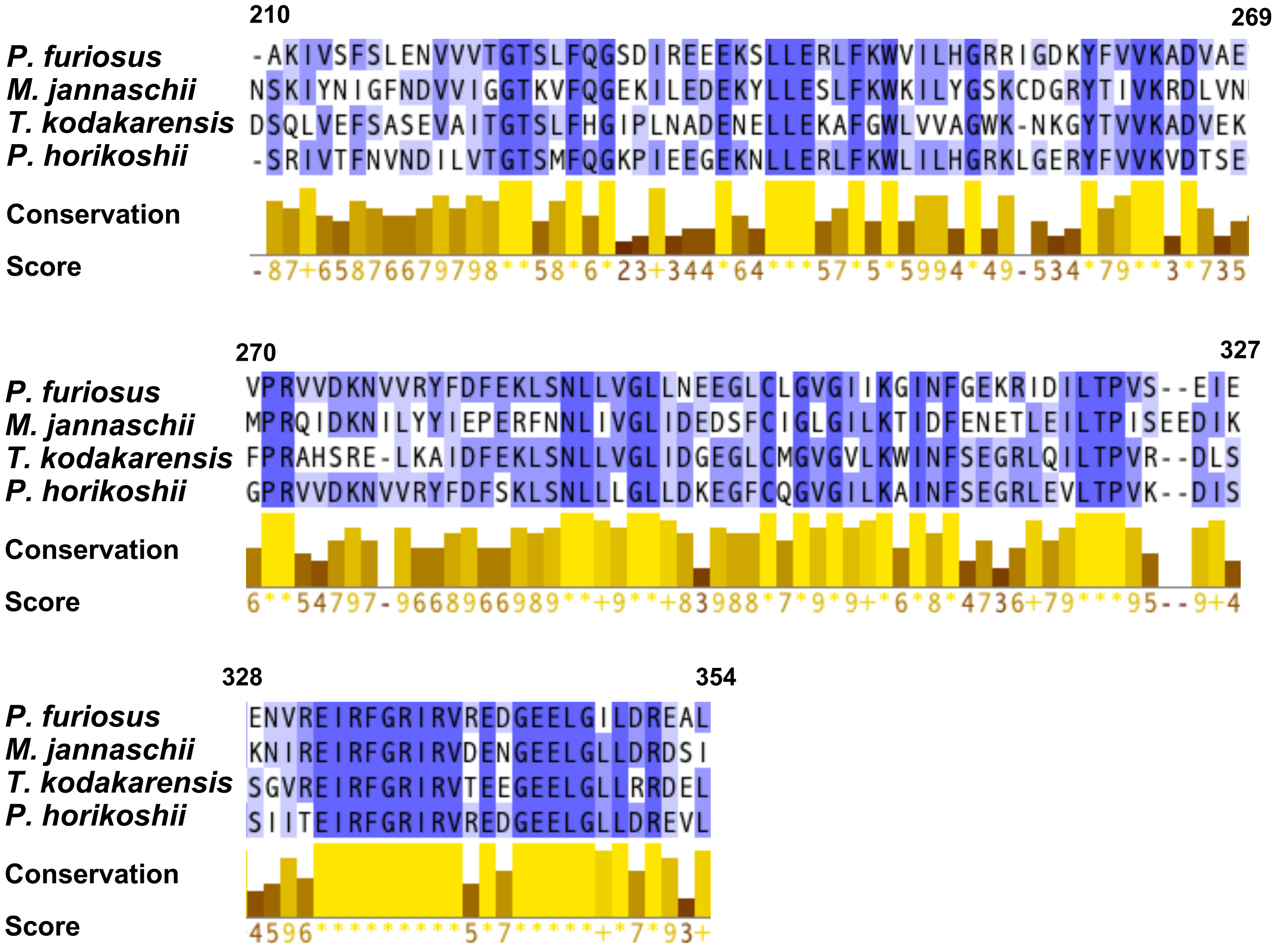

Supplementary Figure S4. Amino acid sequence alignment of Clp1 C-terminal regions (Clp1_aC) from four archaeal species. Amino acid sequences were aligned with MAFFT and the alignments were visualized with Jalview. Identical amino acid residues are indicated in blue and partly conserved amino acid residues are indicated in light blue. Gaps (–) were inserted to maximize the number of amino acid matches. Numbers indicate the positions of the amino acid residues from the first methionine (Met) residue in the *P. furiosus* Clp1 protein (UniProt AC: Q8U4H6). The conservation score for each amino acid position is indicated as one of 12 ranks (0–11). Partially conserved amino acids (rank 10) are shown as a plus symbol, and identical amino acids (rank 11) are indicated with asterisks (Livingstone and Barton 1993). The organisms are: *Pyrococcus furiosus*, *P. horikoshii*, *Methanocaldococcus jannaschii*, and *Thermococcus kodakarensis*.

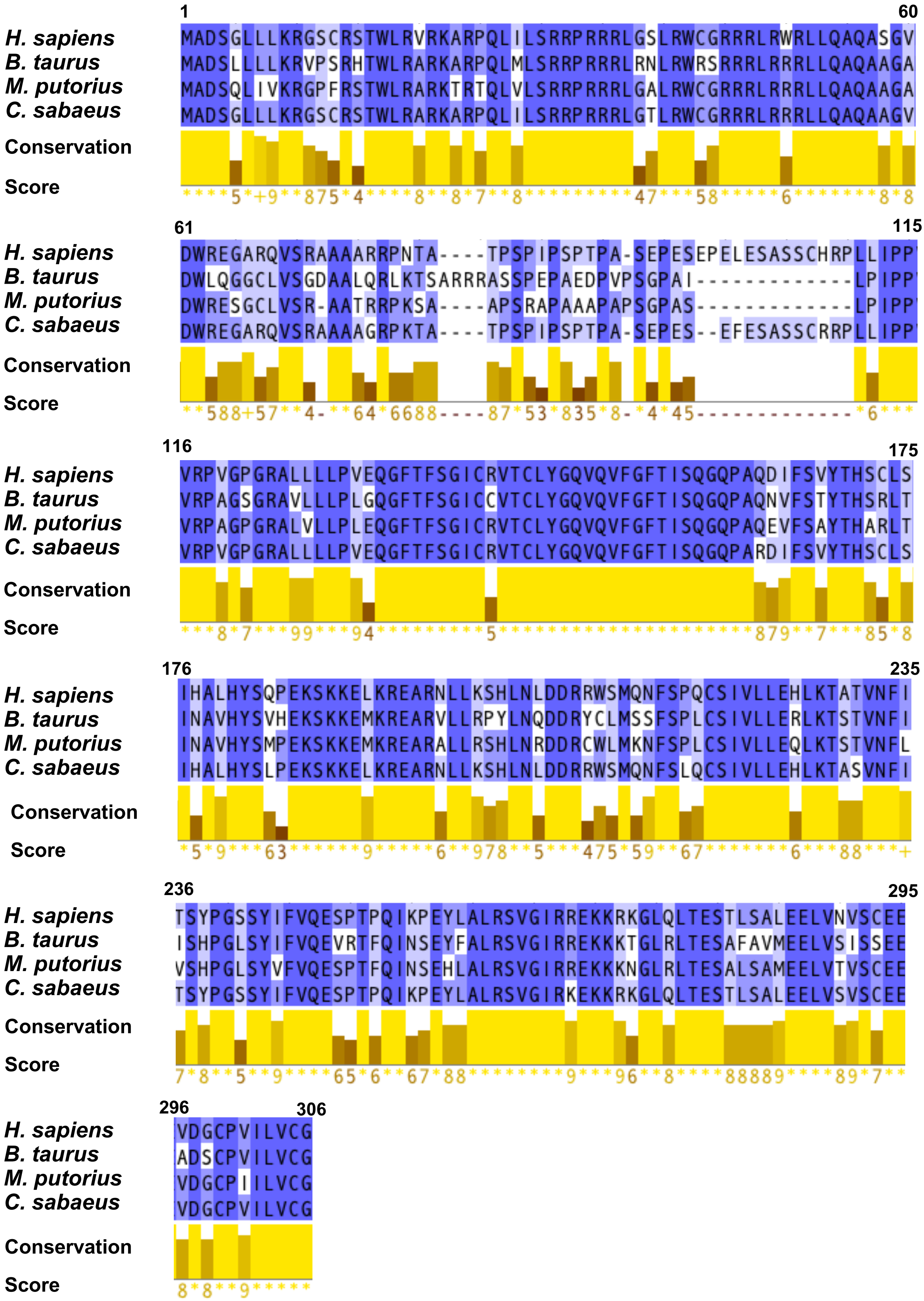

Supplementary Figure S5. Amino acid sequence alignment of Nol9 N-terminal regions (Nol9_eN) from four eukaryote species. Amino acid sequences were aligned with MAFFT and the alignments were visualized with Jalview. Identical amino acid residues are indicated in blue and partly conserved amino acid residues are indicated in light blue. Gaps (–) were inserted to maximize the number of amino acid matches. Numbers indicate the positions of the amino acid residues from the first methionine (Met) residue in the human Nol9 protein (UniProt AC: Q5SY16). The conservation score for each amino acid position is indicated as one of 12 ranks (0–11). Partially conserved amino acids (rank 10) are shown as a plus symbol, and identical amino acids (rank 11) are indicated with asterisks (Livingstone and Barton 1993). The organisms are: *Homo sapiens*, *Bos taurus*, *Mustela putorius*, and *Chlorocebus sabaeus*.

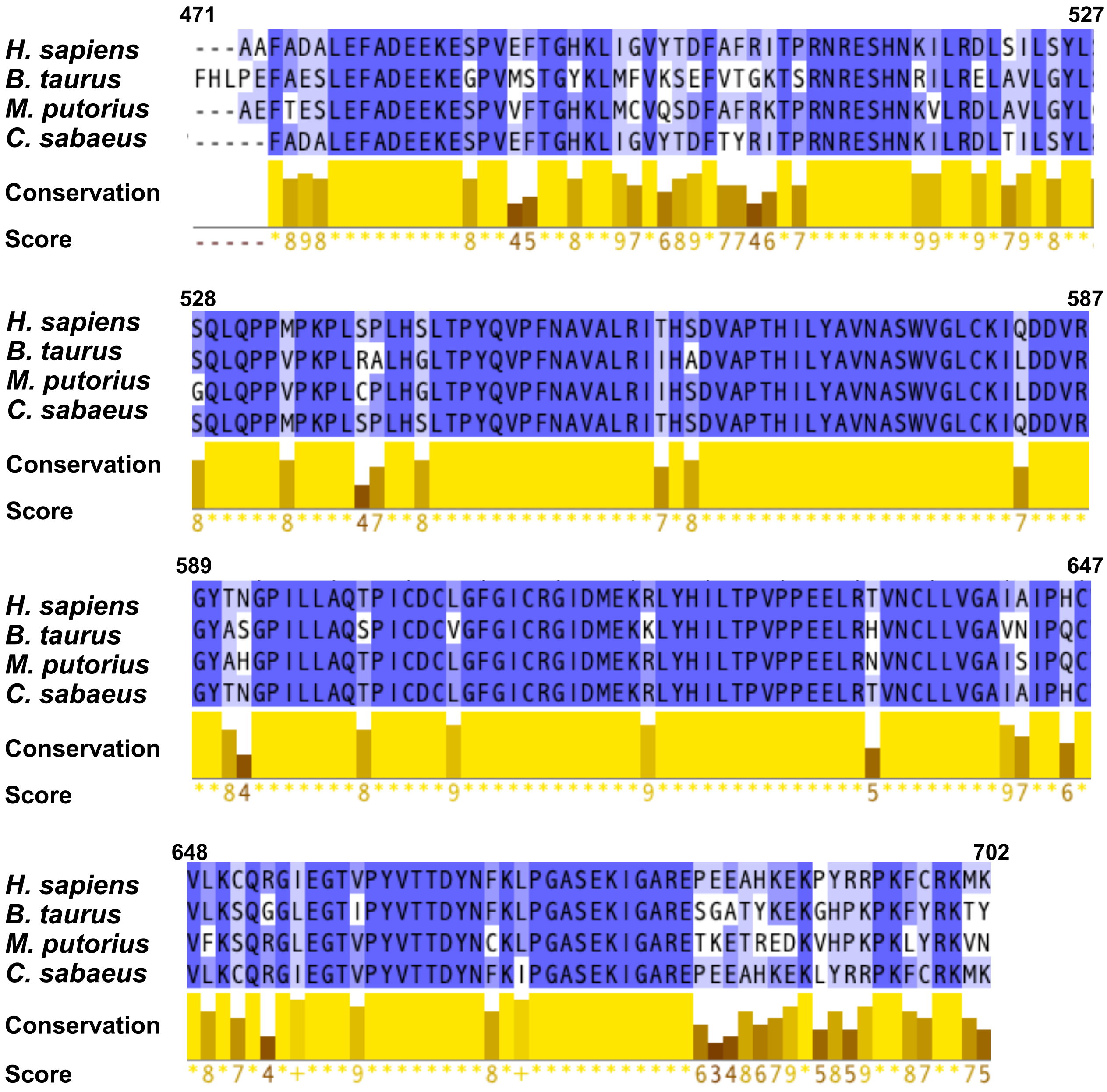

Supplementary Figure S6. Amino acid sequence alignment of Nol9 C-terminal regions (Nol9_eC) from four eukaryote species. Amino acid sequences were aligned with MAFFT and the alignments were visualized using Jalview. Identical amino acid residues are indicated in blue and partly conserved amino acid residues are indicated in light blue. Gaps (–) were inserted to maximize the number of amino acid matches. Numbers indicate the positions of amino acid residues from the first methionine (Met) residue in the human Nol9 protein (UniProt AC: Q5SY16). The conservation score for each amino acid position is indicated as one of 12 ranks (0–11). Partially conserved amino acids (rank 10) are shown as a plus symbol, and identical amino acid (rank 11) are indicated with asterisks (Livingstone and Barton 1993). The organisms are: *Homo sapiens*, *Bos taurus*, *Mustela putorius*, and *Chlorocebus sabaeus*.

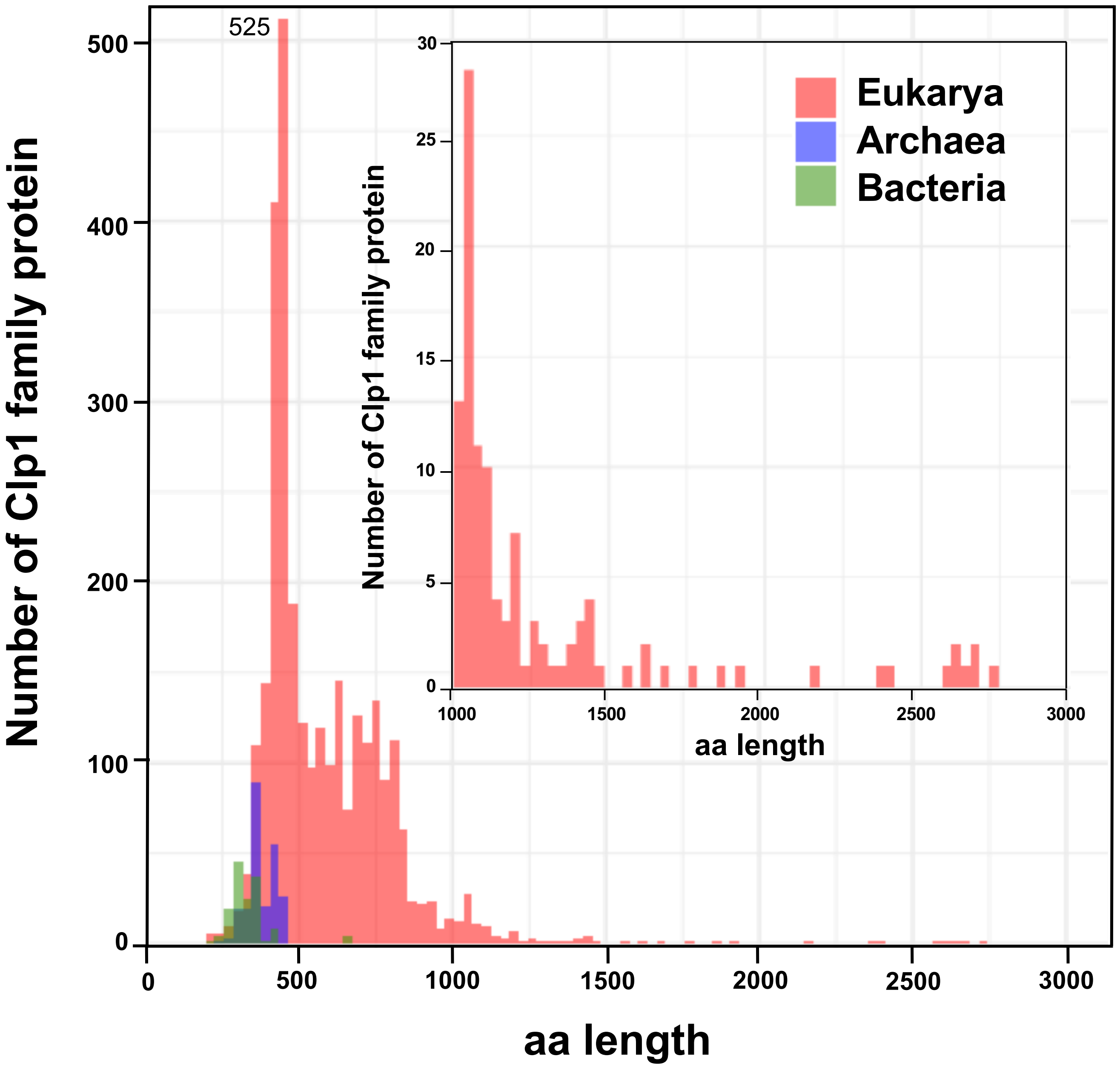

Supplementary Figure S7. Size distribution of Clp1 and Clp1-related proteins. Size distribution of the 3,332 Clp1 and Clp1-related proteins identified in the present study is shown. The vertical axis indicates the number of proteins and the horizontal axis represents the amino acid (aa) length. Enlarged view (aa lengths between 1,000 and 3,000) of the figure is also shown inside. Bacteria (green), Archaea (blue), and Eukarya (red).

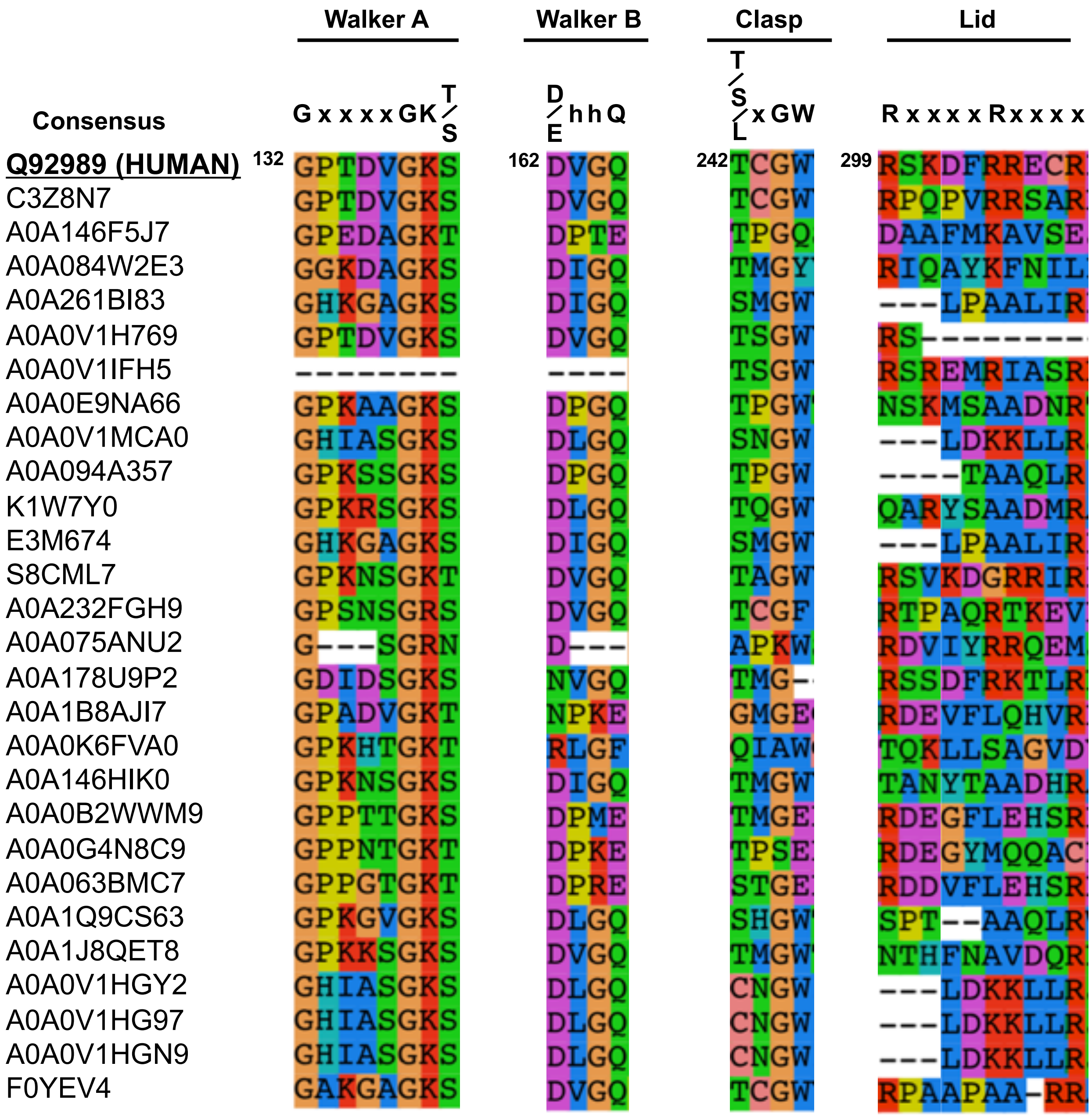

Supplementary Figure S8. Amino acid sequence alignments of the conserved motifs in the polynucleotide kinase domains of the large proteins. Amino acid sequences of the conserved motifs in the polynucleotide kinase domains of the large proteins were aligned with MAFFT and visualized with SeaView. Each color represents the type of amino acid residue. Amino acid numbers from the first methionine (Met) residue of human Clp1 protein (UniProt AC: Q92989), are shown on the left of each line.

```
Original   1 ATGCTCCTCCTGGCTGGCCCCACGGATACCGGCAAGTCCACCCTGGCCTTAAGGCTTCTG  60
             *** * *** ******* ***** ******** ** ** ** ****** *  *  * ***
Optimized  1 ATGTTACTCTTGGCTGGACCCACCGATACCGGGAAATCGACACTGGCCCTGCGTTTGCTG  60

          61 GAAAAGGCCAAGGAAGCCTACCTCCTGGACCTGGACCCGGGGCAGGGAGCCCTGCCCGGG 120
             ** ** ** ** ***** ** *********** ** ** ** ***** *** * ** **
          61 GAGAAAGCGAAAGAAGCGTATCTCCTGGACCTTGATCCTGGACAGGGCGCCTTACCGGGT 120

         121 GCCTTCACCCTCTTCCACTACCGGGAAGGAACCCTCACCCCCCTACGCCGCTACCTTCTG 180
             ** ****** * ***** ***** ***** ** ** ** **  *************  **
         121 GCTTTCACCTTGTTCCATTACCGTGAAGGCACGCTGACGCCGTTACGCCGCTACCTGTTG 180

         181 GGGGCCCTGTCCCCCAGAGGGATGGAGGCGCAGGCGGTGGTGGGGGCCCTGCGCCTAGCC 240
             ** *** **    **  * ** ***** ** ** ** ***** ***** ******** **
         181 GGCGCCTTGAGTCCGCGTGGTATGGAAGCACAAGCCGTGGTTGGGGCACTGCGCCTTGCG 240

         241 CGCCTCATCCCCAAAGGAAGCCCCGCCGTGGCCGACACGGATGGCTACCTGGACCCTGGG 300
             ** ** ** ** *****   *** ** ** ** ***** ********* * ** ** ***
         241 CGTCTGATTCCGAAAGGCTCCCCAGCGGTAGCTGACACCGATGGCTACTTAGATCCCGGG 300

         301 TTTCGGCTCTTACAGATCGAGGCCCTGGTGCCCGCCGAGGTGCTGGTCCTGGGGTGGGAG 360
             ***** **  * ******** ***** ** ** ***** ******** ***** *****
         301 TTTCGCCTGCTGCAGATCGAAGCCCTTGTTCCTGCCGAAGTGCTGGTACTGGGCTGGGAA 360

         361 GAACTCTACCAGGCCCTTTCCTGGCGCAGGGACCTAAGGGCGCGCCTGGCCCCGCCCCTT 420
             ** ** ** ***** **   ******  ***** ** * ** ******** ** ** **
         361 GAGCTGTATCAGGCGCTGAGCTGGCGTCGGGACTTACGTGCACGCCTGGCGCCTCCGCTC 420

         421 CAGGGGGTCCGCAGGAAAACCCCGGCCGAGAGACGGAAAAACCGCCTGGAAAGGCTTTTT 480
             ** ** ** *** * ***** ***** **  * ** ** ************ * ** **
         421 CAAGGTGTGCGCCGCAAAACGCCGGCTGAACGCCGCAAGAACCGCCTGGAACGCCTGTTC 480

         481 GCCCACTTCCAGGAAGCCAGGCCCCGTCCCCTTCCCCTCGGTCACATGCCCGCATACCCT 540
             ** ** ** ***** **  * ** ***** ** *** * ** ******** ** ** **
         481 GCGCATTTTCAGGAGGCACGTCCGCGTCCACTGCCCTTGGGACACATGCCAGCGTATCCG 540

         541 ATGGAACCCCCCGAGCCCCAGCGGCTTTACGGGCTCCTGGACGGGGAAGGGTTCCTCCTG 600
             ******** ** ** ** ******** ** ** ** ** ** ** ** ** ** ** **
         541 ATGGAACCTCCGGAACCACAGCGGCTGTATGGTCTGCTTGATGGTGAGGGTTTTCTGCTC 600

         601 GGCTACGGACGGCTTCTGGCCTGGGCAGGGGACGAGGGACTTTTCCTCACCCCCGTGGGG 660
             ***** ** **  * ***** ******** ** ** ** ** ** ** ** ** ** **
         601 GGCTATGGCCGCTTACTGGCGTGGGCAGGCGATGAAGGTCTGTTTCTGACTCCGGTTGGC 660

         661 GAGGAGGTGGCCAGGGTGGTACCCACCCGGCTTCTTTTCCCTATCCCCGCACTACCAGGT 720
             ** ***** *** * ***** ** ** ** ** ** ** ** ** ** ***** ** ***
         661 GAAGAGGTCGCCCGTGTGGTCCCAACTCGCCTGCTCTTTCCGATTCCGGCACTTCCGGGT 720
```

Supplementary Figure S9. Comparison of the original and codon-optimized sequences of *Ts*-Clp1. For the efficient expression of the recombinant *Ts*-Clp1 protein (UniProt AC: E8PQM6) in *Escherichia coli*, the synthetic gene was designed to optimize codon usage. Changed nucleotides are boxed. These two genes encoded exactly the same polypeptide.

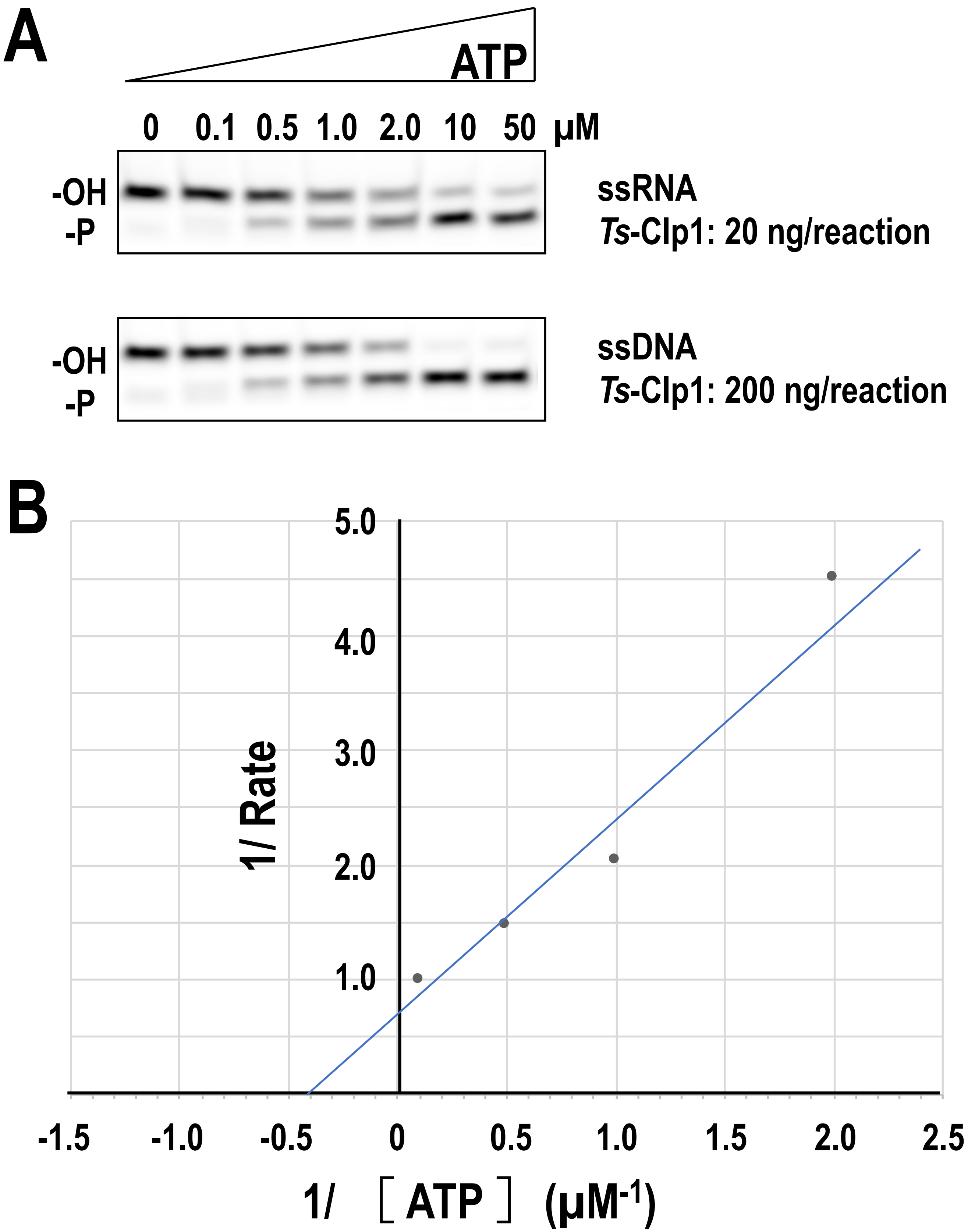

Supplementary Figure S10. Determination of the Km value of *Ts*-Clp1 for ATP. (A) Effect of ATP concentration on the polynucleotide kinase activity of *Ts*-Clp1. A 3′-fluorescein amidite (FAM)-labeled oligoribonucleotide was incubated with purified *Ts*-Clp1 protein (either 1.0 µg/mL for ssRNA or 10 µg/mL for ssDNA) and 10 mM $MgCl_2$, together with various concentrations of ATP, at 60 ° C for 15 min. The products were separated by 15% polyacrylamide gel electrophoresis with 8 M urea. The reaction products were visualized and quantified with Molecular Imager FX Pro. (B) Lineweaver–Burk plot of the data from A (ssRNA). The *K*m value for ATP was determined to be 2.5 µM when the ATP concentrations were varied in the range 0.5–10 µM.

Reference for Supplementary Information:

Livingstone CD and Barton GJ. 1993. Protein sequence alignments: a strategy for the hierarchical analysis of residue conservation. Comput Appl Biosci 9(6): 745-756.
